# Respiratory Outbreak Mitigation With Point-of-Care Testing in Long-Term Care: A Randomized Clinical Trial

**DOI:** 10.1001/jamainternmed.2026.2644

**Published:** 2026-07-06

**Authors:** Christopher Kandel, Daphne Oriotis, Heather L. Candon, James Callahan, Christina K. Chan, Aaron Truong, Alexia Rookwood, Stephen Suleiman, Carla Rosario, Brian M. Wong, Shawn J. Palmer, Brigitte Pascual, Victoria Serapion, Lisa Marcovici, Sid Feldman, Paul M. Yip, Robert A. Kozak, Tracy Taylor, Paul Sanstrom, Kathleen Kirk, Justin Eplett, Radhika Chawla, Kevin A. Brown, Ian Brasg, Jeff Powis, Jerome A. Leis

**Affiliations:** 1 Michael Garron Hospital, Toronto, Ontario, Canada; 2 Dalla Lana School of Public Health, Toronto, Ontario, Canada; 3 Temerty Faculty of Medicine, University of Toronto, Toronto, Ontario, Canada; 4 Sunnybrook Health Sciences Centre, Toronto, Ontario, Canada; 5 Humber River Health, Toronto, Ontario, Canada; 6 Baycrest Health Sciences, Toronto, Ontario, Canada; 7 Centre for Quality Improvement and Patient Safety, University of Toronto, Toronto, Ontario, Canada; 8 JC Wilt Infectious Diseases Research Centre, National Microbiology Laboratory, Winnipeg, Manitoba, Canada

## Abstract

**Importance:**

Respiratory infections caused by SARS-CoV-2, influenza, and respiratory syncytial virus (RSV) cause pronounced seasonal morbidity and mortality among residents of nursing homes (NHs).

**Objective:**

To assess the effect of an on-site point-of-care respiratory multiplex polymerase chain reaction (POC-RMPCR) instrument on NH outbreaks.

**Design, Setting, and Participants:**

This multicenter, open-label cluster randomized trial was conducted from November 12, 2024, to May 2, 2025, in NHs in Toronto, Ontario, Canada. The data analysis was performed from October 7, 2025, until December 31, 2025.

**Intervention:**

On-site POC-RMPCR by trained NH staff.

**Main Outcomes and Measures:**

The primary outcome was jointly SARS-CoV-2, influenza, and RSV outbreak size and number, and secondary outcomes included the rates of resident emergency department (ED) transfer and death.

**Results:**

Among 20 participating NHs with a total of 3963 beds, there was median of 5.5 units, with 30 beds per unit and a crowding index score of 1.42. The joint estimate of outbreak number (51 and 62, respectively) and size for the intervention group was no different from controls, with a rate ratio of 1.12 (95% CI, 0.78 to 1.58). ED transfers for confirmed (−3.5%; 95% CI, −7.2 to −0.2%) and confirmed/suspected infection (−11.0%; 95% CI, −20.6% to −2.0%) were lower among intervention NHs without a difference in death. Viral testing rates (3.69 tests/week vs 1.73 tests/week) and ratio of confirmed to suspect cases (4.2 vs 2.0) were higher in intervention homes along with a shorter time to initiation of antiviral therapy from symptom onset (−2.5 days; 95% CI, −3.1 to −1.9).

**Conclusions and Relevance:**

The results of this cluster randomized clinical trial suggests that use of a POC-RMPCR in NHs did not change outbreak number or size but decreased the number of ED transfers in the context of increased viral testing, improved case detection, and faster initiation of antiviral therapy for influenza. Seasonal adoption of POC-RMPCR in NHs would avoid an estimated 4 ED transfers per 100 beds.

**Trial Registration:**

ClinicalTrials.gov Identifier: NCT06660433

## Key Points

**Question:**

Does point-of-care respiratory multiplex polymerase chain reaction (POC-RMPCR) testing in nursing homes (NHs) lead to smaller outbreak size or better resident outcomes due to SARS-CoV-2, influenza, and respiratory syncytial virus infection?

**Findings:**

In this cluster randomized trial of 20 NHs, use of an on-site POC-RMPCR instrument did not decrease outbreak number or size. POC-RMPCR enabled increased testing, improved case detection, and shorter time to initiation of antiviral therapy from symptom onset (influenza only).

**Meaning:**

The trial results suggest that seasonal adoption of POC-RMPCR in NHs would avoid an estimated 4 hospital transfers per 100 beds, equivalent to 64 000 across the US each respiratory season.

## Teaser

This cluster randomized trial examines the effect of an on-site point-of-care respiratory multiplex polymerase chain reaction instrument on outbreaks of SARS-CoV-2, influenza, and respiratory syncytial virus in nursing homes.

## Introduction

Respiratory infections caused by SARS-CoV-2, influenza, and respiratory syncytial virus (RSV) cause pronounced seasonal morbidity and mortality among residents of nursing homes (NHs).^1,2,3,4,5^ A key lesson from the COVID-19 pandemic was the importance of robust infection prevention and control (IPAC) programs in NHs.^1,6^ Earlier detection of residents infected with SARS-CoV-2, influenza, and RSV attenuates outbreak size and resident complications.^6,7,8^

Effective respiratory virus surveillance in an NH depends on prompt symptom recognition and viral testing results. Despite improvements to syndromic surveillance, test turnaround time (TAT) from specimen collection to the results of respiratory multiplex polymerase chain reaction (RMPCR) takes days due to specimen transport and processing at a reference laboratory.^9,10,11^ A national survey of more than 15 000 NHs in the US found that 45% had a TAT of 3 days or longer.^11^ A protracted TAT contributes to delays in implementation of control measures. The median time to receipt of antiviral therapy for residents with influenza, a recognized predictor of influenza outcomes among older adults, exceeds 3 days among care homes in the UK.^12,13^

Point-of-care (POC) respiratory virus testing in NHs has the potential to mitigate the effect of respiratory outbreaks through enhanced IPAC and clinical management. A randomized clinical trial showed that rapid influenza testing with an immunoassay, which is less sensitive than PCR, reduced hospitalizations among NH residents.^14^ Rapid antigen tests (RATs) introduced in NHs during the COVID-19 pandemic also require confirmatory molecular testing.^15^ POC-RMPCR instruments provide results for multiple viral respiratory targets within 35 minutes, with comparable sensitivity to reference laboratories.^16,17,18^ While these lead to reduced time to initiation of precautions and antiviral therapy, to our knowledge, the effect on NH outbreaks has never been evaluated.^19,20^ In addition, using a POC-RMPCR in NHs is challenged by questions of feasibility due to staff workload and increased costs.^17,18,21,22^

In a recent pilot study in Toronto, Ontario, Canada, we found that trained NH staff could effectively use a POC-RMPCR instrument, resulting in a marked reduction in TAT and time to detection of outbreaks.^10^ The objective of the following cluster randomized clinical trial was to evaluate whether use of a POC-RMPCR instrument could reduce outbreak size and improve resident outcomes during the 2024 to 2025 respiratory season.

## Methods

### Trial Design

The imProving Respiratory Outbreak Mitigation Through Point-of-Care Testing in Long-Term Care (PROMPT-LTC) trial is a multicenter, open-label, cluster randomized clinical trial comparing the use of a POC-RMPCR instrument with standard of care (transferring respiratory specimens to a local reference microbiology laboratory for testing). All facilities affiliated with a hospital-based team in the east, north, and northwest regions of Toronto, Ontario, Canada, were eligible to participate. The study was conducted from November 12, 2024, until May 2, 2025. The data analysis was performed from October 7, 2025, until December 31, 2025. The research ethics board of Michael Garron Hospital approved the study and waived informed consent, with each home agreeing to participate. The trial protocol and statistical analysis plan are available (Supplement 1 and Supplement 2), and the study was registered before initiation (NCT06660433). Reporting of the results of the trial followed the Consolidated Standards of Reporting Trials (CONSORT) reporting guidelines.^23^

### Randomization and Masking

Participating NHs were randomized in a 1:1 allocation to receive a POC-RMPCR instrument (Cepheid GeneXpert Xpress System using Xpress CoV-2/Flu/RSV plus cartridge) vs standard of care. Homes were matched by mean crowding index scores and scaled bed size and randomized centrally using a computer-generated sequence that was overseen by the trial statisticians. The trial was open label, with analysis performed by masked statisticians who were unaware of allocation arm.

### Participating Facilities

The care of NH residents and resources provided to support IPAC and clinical care was the same irrespective of group allocation. Standardization of clinical practices and IPAC measures was encouraged through a virtual community of practice (COP) composed of representatives from all NHs. The clinician COP met weekly during November and December 2024 to ensure a standardized approach to clinical care of residents with SARS-CoV-2, influenza, or RSV infection. Similar policies to accessing antiviral therapy were present across NHs, including preauthorized use of oseltamivir for influenza and mechanisms to arrange nirmatrelvir/ritonavir or intravenous remdesivir for residents for symptomatic SARS-CoV-2, with dexamethasone reserved for those who required supplemental oxygen. The IPAC lead of each NH participated in a separate weekly virtual COP that was facilitated by hospital IPAC leaders. It focused on heightened surveillance and timely initiation of precautions, contact tracing, and use of oseltamivir prophylaxis. Outbreaks were managed by NH facilities according to local seasonal public health guidelines, for which PCR testing was expected to be performed for all symptomatic residents, including throughout outbreaks. Aside from the testing instrument, the difference in IPAC protocols was that intervention homes performed testing of high-risk close contacts of residents with SARS-CoV-2 infection on day 3 from the date of exposure; otherwise testing was reserved for symptomatic residents only in both study arms. RATs for SARS-CoV-2 remained available in some control NHs but all respiratory specimens regardless of RAT results were submitted to nearby reference laboratories for expanded RMPCR (9 targets, including SARS-CoV-2, influenza A, influenza B, RSV, adenovirus, parainfluenza, rhino/enterovirus, human coronavirus, and human metapneumovirus). The intervention group relied exclusively on POC-RMPCR but forwarded the specimen to a reference laboratory for expanded RMPCR testing (same as control sites) if 2 or more residents in the unit were symptomatic and had negative test results. There were no costs to NHs for using RMPCR, regardless of allocation group.

### Implementation of POC-RMPCR

Between October 14, 2024, and November 7, 2024, the NHs randomized to the intervention received training on how to use the POC-RMPCR instrument, as previously described.^10^ NH staff (ie, registered nurse, IPAC lead) received a 1-hour session on how to operate the instrument, avoid cross-contamination, store specimens, and perform quality control (QC). Each trained staff member was certified after successfully processing a specimen independently and passing a knowledge test. Each NH determined the number of staff to train and hours of operation. On receiving the POC-RMPCR instrument, each NH conducted a panel of 8 external QC tests to validate the instrument along with 1 external masked QC (verified by the National Microbiology Laboratory [Winnipeg, Manitoba, Canada]).

### Data Collection and Follow-Up

Baseline characteristics of each participating NH were collected, including crowding index scores, bed numbers, for-profit status, and receipt of resident seasonal vaccinations against influenza and SARS-CoV-2. Residents were considered a confirmed case of SARS-CoV-2, influenza, or RSV with a positive RMPCR test result, whether from POC-RMPCR, reference laboratory, or if tested in the emergency department (ED). To minimize the effect of missing outcomes due to differential testing, suspected cases were defined as a resident of an outbreak unit who did not have a RMPCR-confirmed positive test result but died or were transferred to a hospital ED between the start of an outbreak and up to 14 days from the symptom onset of the last confirmed case. High-risk contacts were prospectively identified by IPAC leads and defined as a resident sharing a room or adjoining washroom or any close interaction with a source resident in a small or poorly ventilated room.^24,25^ For each infected resident, the date of symptom onset and antiviral initiation was recorded. The outcomes of each resident within 28 days of symptom onset were separated into those who recovered in the NH, transferred to ED and recovered, died, and died after transfer to the ED. To assess testing volumes, a convenience subset of homes (8 NHs located in the East Toronto region) counted all viral tests that were performed.

### Outcomes

The primary outcome was jointly the number and size of SARS-CoV-2, influenza, and RSV outbreaks at the unit level. Each outbreak was defined as 2 or more residents with the same confirmed viral infection within a 14-day period on a unit. This definition was broader than the provincial definition of 2 or more cases within 7 days to minimize unrecognized outbreaks.^26^ Outbreaks were considered over when no additional confirmed cases were identified for 14 days from symptom onset of the last confirmed case. Outbreak size was defined as the number of confirmed or suspected cases from the start to the end of an outbreak. Secondary outcomes included outbreak length (the number of days between the first and last identified case), number of confirmed or suspected secondary resident infections following introduction of a confirmed case in a unit, number of residents who transferred to the ED or died within 28 days, and time from symptom onset to initiation of antiviral therapy.

### Statistical Analysis

Baseline characteristics were compared between the intervention and control homes. The sample size was estimated based on simulation using the mean bed size of NHs in Ontario, Canada (n = 120), and an average of 4.8 introductions of a respiratory virus per year, with 50% progressing to an outbreak. For 24 NHs, the expected power was 0.81, with an assumed effect size of 40%. Prior to randomization, 4 NHs withdrew, reducing the power to 0.73. The primary outcome was the risk of outbreak-associated cases, which was estimated using a joint model of the outbreak number and secondary case size. The number of outbreaks was modeled by fitting a Poisson model, with virus and allocation included as fixed covariates and NH as the random effect while outbreak size was modeled by fitting a binomial model with the number of secondary cases and noncases in each outbreak. The effect size of the intervention was jointly modeled using the metafor package, version 4.8-0. Transfer and deaths were modeled by fitting a logistic regression model that was adjusted for the same variables, while time from symptom onset to antiviral initiation was modeled using a linear regression. Subgroup analyses included SARS-CoV-2 and influenza outbreaks separately. Absolute risk differences between groups and conditional ratios were presented. No correction for multiplicity was applied. Weekly testing volumes were compared by allocation groups for the convenience sample of East region NHs. The rate of combined ED transfer or death per 100 beds between groups was compared among residents with confirmed respiratory infection, suspected/confirmed infection, outbreak/nonoutbreak units, and overall. All analyses were conducted using R (version 4.4.3; R Foundation) using the glmmTMB and lme4 packages.

## Results

**Figure 1.**
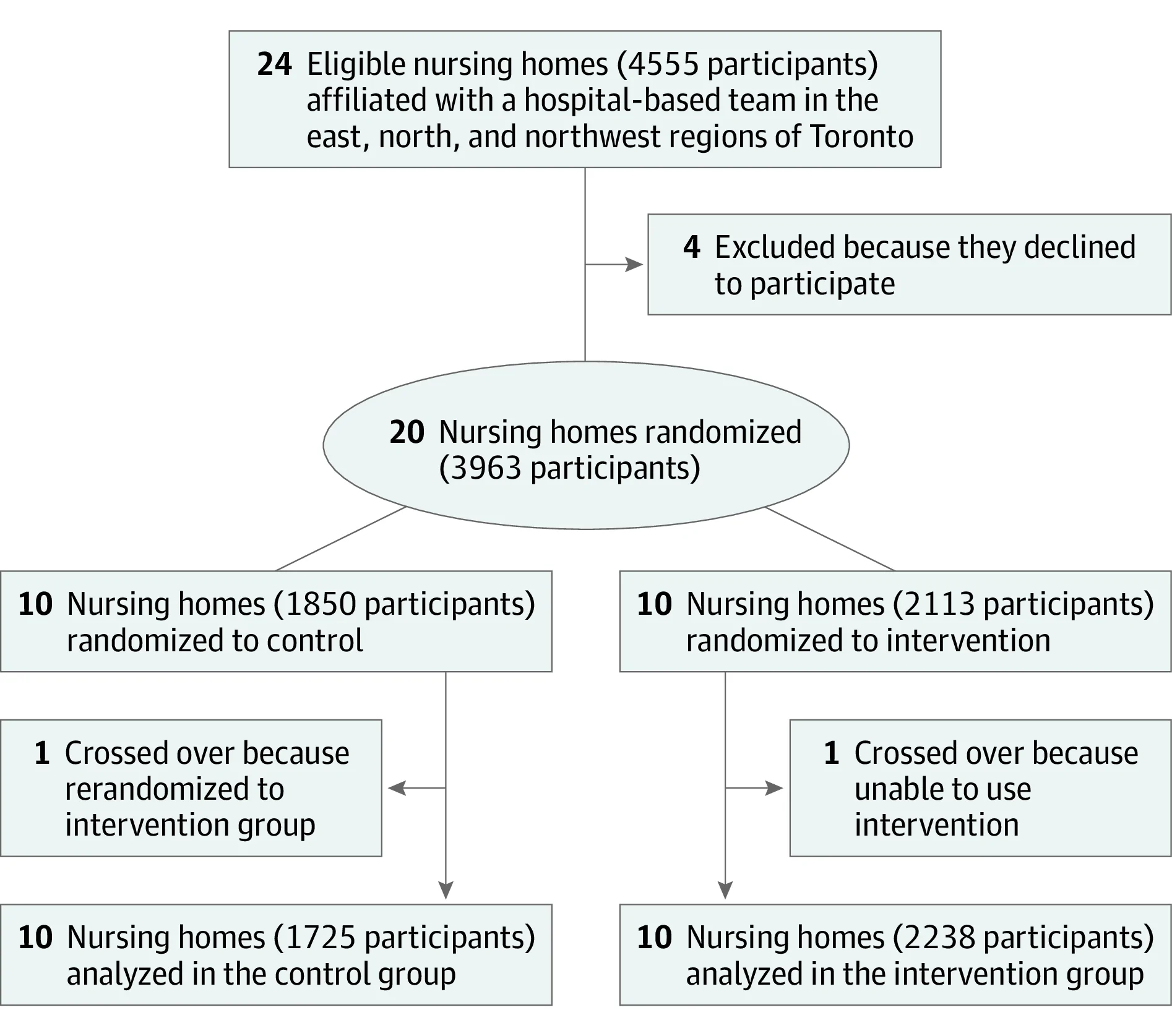
CONSORT Flow Diagram of a Cluster Randomized Trial of Nursing Homes Using Point-of-Care Respiratory Multiplex Polymerase Chain Reaction Testing vs Standard Respiratory Multiplex Polymerase Chain Reaction at an Offsite Local Laboratory

Among the 24 eligible NHs, 20 NHs (83.3%) participated, with 10 in the control arm and 10 in the intervention arm (Figure 1; eTable 1 in Supplement 3). After randomization and before study initiation, 1 NH that was allocated to the intervention group was unable to support implementation and substituted with a randomly selected home from the control group to ensure all instruments were used.

On average, each NH was composed of 6 units, with approximately 30 beds per unit (Table 1) (median of 11 staff per NH; IQR, 6-17). Resident vaccination rates were balanced between the groups while there was slightly higher crowing index score, larger facility size, and more for-profit NHs in the intervention group. Internal weekly QC results reported by facility IPAC leads were all valid (216 of 220 [98.1%]).

**Table 1.** Characteristics of the Participating Nursing Homes

Characteristic	Overall (N = 20)	Control (n = 10)	Intervention (n = 10)	Standardized mean difference
Total beds, No.	3963	1725	2238	NA
Units per home, median (IQR)	5.5 (4.0-8.2)	5.0 (3.2-6.8)	6.5 (4.2-9.5)	0.58
Beds per unit, median (IQR)	30.0 (26.0-32.0)	30.5 (26.0-32.0)	28.0 (25.5-33.0)	0.19
Crowding index score, mean (SE)	1.42 (0.04)	1.34 (0.06)	1.47 (0.06)	0.11
Location, No. of homes				
East	8	4	4	0
North	4	2	2	
Northwest	8	4	4	
Profit status, No. of home				
For profit	7	2	5	0.63
Nonprofit	13	8	5	
Resident SARS-CoV-2 vaccination, %				
Yes	44.8	48.8	41.1	0.16
No	55.2	51.2	58.9	
Missing	0	0	0.1	
Resident influenza vaccination, %				
Yes	67.2	66.9	67.5	0.05
No	32.8	33.1	32.5	
Missing	0	0	0.1	

Abbreviation: NA, not applicable.

There were 937 cases overall, including 695 residents with a confirmed respiratory virus infection by RMPCR, along with 242 suspected infections that included residents without a positive test result who were transferred to an ED or died during an outbreak (eTable 2 in Supplement 3). The ratio of confirmed to suspected cases was more than twice higher in the intervention group (4.2 vs 2.0). Testing of asymptomatic exposed residents accounted for 40 of 695 confirmed cases (6%): 36 in the intervention group and 4 in the control group. In addition to SARS-CoV-2, influenza, and RSV, there were 27 PCR-confirmed infections (3.4%) with other respiratory viruses, including 9 in control homes and 18 in intervention homes.

**Figure 2.**
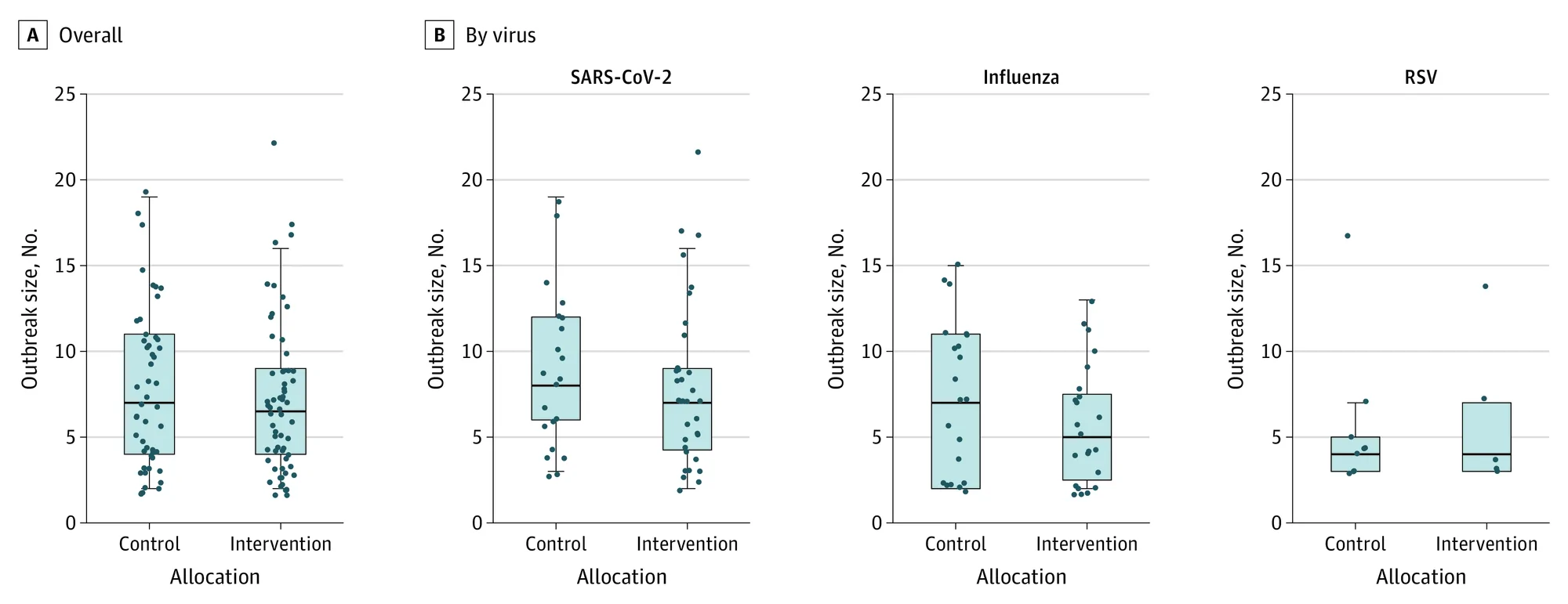
Box-and-Whisker Plots of Outbreak Numbers and Sizes by Allocation Group On-site point-of-care respiratory multiplex polymerase chain reaction instrument vs standard off-site testing for all homes (A) and separated by virus (B). Each allocation group is presented as a box plot with the median and interquartile ranges with the size of each outbreak overlaid. RSV indicates respiratory syncytial virus.

There were 113 viral respiratory outbreaks: 51 in the control group and 62 in intervention NHs (Figure 2). Outbreaks of SARS-CoV-2 were the most frequent, with a median of 7 (IQR, 4-11) cases in the control group and 6.5 (IQR, 4-9) in the intervention group (eTables 3-4 in Supplement 3).

The primary outcome of the joint estimate of the outbreak number and size for the intervention group was no different from the control group, with a rate ratio of 1.12 (95% CI, 0.78 to 1.58; *P* = .55). The effect of the intervention was a decrease in resident ED transfers due to confirmed infection (absolute difference, −3.5%; 95% CI, −7.2 to −0.2%) and was more pronounced when both suspected or confirmed infection were included (−11.0%; 95% CI, −20.6% to −2.0%; Table 2). This difference equates to 4 fewer ED transfers per 100 beds. In a subgroup analysis by virus, avoided ED transfers were driven by SARS-CoV-2 (absolute difference, −19.5%; 95% CI, −32.0% to −7.0%) as compared with influenza (absolute difference, −2.7%; 95% CI, −17.0% to 11.6%; eTables 5-7 in Supplement 3).

**Table 2.** Secondary Outcomes Comparing Control and Intervention Nursing Homes as Stratified by Confirmed and Confirmed/Suspected Viral Respiratory Infection

Outcome	Measure (95% CI)				*P* value
	Control	Intervention	Absolute difference (intervention − control)	OR	
**Confirmed viral respiratory infection**					
Outbreak size (secondary cases)	2.91 (2.47 to 3.40)	3.77 (3.40 to 4.15)	0.86 (0.26 to 1.45)	1.53 (0.84 to 2.79)	.16
Proportion of secondary cases per outbreak	0.68 (0.57 to 0.79)	0.69 (0.61 to 0.78)	0.01 (−0.11 to 0.13)	1.06 (0.53 to 2.10)	.87
Transfer	0.11 (0.07 to 0.15)	0.06 (0.04 to 0.09)	−0.04 (−0.07 to 0)	0.52 (0.29 to 0.96)	.04
Death	0.02 (−0 to 0.04)	0.05 (0.01 to 0.09)	0.02 (−0.01 to 0.04)	2.79 (0.63 to 12.25)	.18
**Confirmed and suspected viral respiratory infection**					
Outbreak size (secondary cases)	5.49 (4.78 to 6.29)	5.38 (4.93 to 5.83)	−0.12 (−0.99 to 0.78)	1.17 (0.75 to 1.84)	.50
Proportion of secondary cases per outbreak	0.79 (0.72 to 0.86)	0.76 (0.70 to 0.82)	−0.02 (−0.10 to 0.06)	0.86 (0.50 to 1.48)	.58
Transfer	0.36 (0.27 to 0.46)	0.23 (0.16 to 0.29)	−0.11 (−0.21 to −0.01)	0.50 (0.29 to 0.88)	.02
Death	0.07 (0.02 to 0.13)	0.09 (0.04 to 0.15)	0.02 (−0.06 to 0.11)	1.27 (0.49 to 3.28)	.63

**Figure 3.**
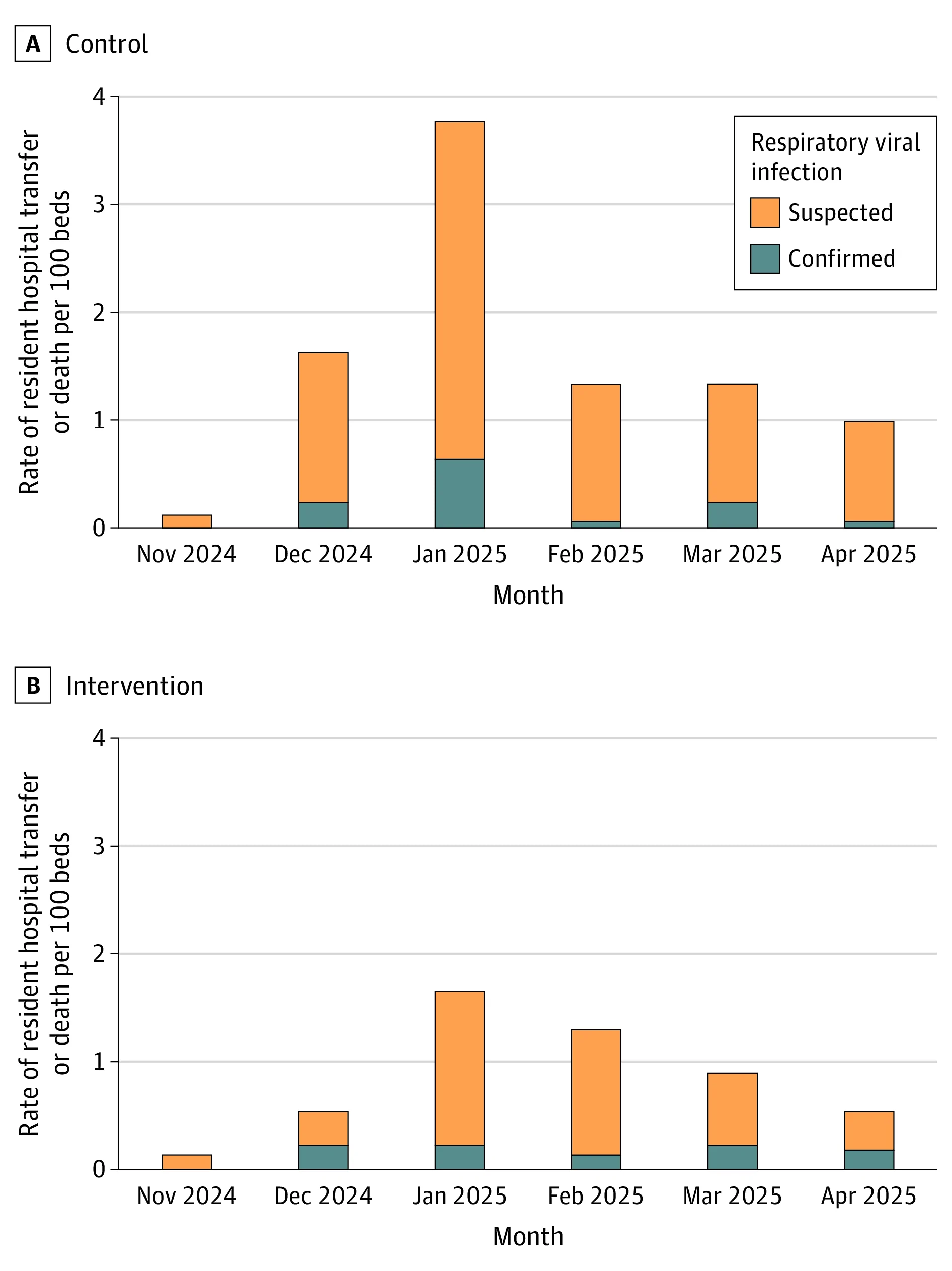
Bar Graphs of Monthly Rates of Resident Emergency Department (ED) Transfer or Deaths per 100 Beds During Outbreaks by Allocation Group A confirmed viral respiratory infection denotes a nursing home resident with a confirmed polymerase chain reaction (PCR) test for SARS-CoV-2, influenza, or respiratory syncytial virus. A suspected case is a resident without a positive PCR test result who resides on the outbreak unit and either died or was transferred to hospital ED during and up to 14 days from the last confirmed outbreak case.

Compared with the control group, the rates of combined hospital ED transfer or death per 100 beds was attenuated in the intervention group during peak viral respiratory season months (December and January) for residents with confirmed and suspected viral respiratory infection (Figure 3). The same seasonality was observed for overall hospital ED transfer or death per 100 beds, with an overall 28% lower rate in the intervention group (eFigure 1 in Supplement 3). The rate of resident ED transfers or death in nonoutbreak units was slightly higher in control NHs, and this difference was magnified on outbreak units (eTable 8 in Supplement 3).

Antivirals were more commonly provided for residents with influenza (84%) compared with SARS-CoV-2 (59%). Time to antiviral therapy from symptom onset was faster in the intervention homes for influenza (0.5 days [95% CI, 0.01-0.9 days] vs 2.9 days [95% CI, 2.5-3.3 days]) but not for SARS-CoV-2 infection (eFigure 2 and eTable 9 in Supplement 3). In the subset of NHs where all viral tests counted, the testing rate was more than 2-fold higher in intervention homes (3.69 tests/week [39% positive] vs 1.73 tests/week [75% positive]; eTable 10 in Supplement 3).

## Discussion

In this cluster randomized clinical trial of 20 NHs in Toronto, Ontario, Canada, use of POC-RMPCR did not decrease outbreak number or size but resulted in an 11% absolute reduction in the probability of ED transfer during an outbreak of SARS-CoV-2, influenza, or RSV. The improved detection of secondary transmission due to increased testing, along with a faster time to initiation of antiviral therapy for influenza, likely explains these findings.

Outbreak number and size was the primary outcome based on the assumption that testing volumes would be similar between groups and the expectation of smaller outbreak sizes, with faster detection as supported by previous observational studies.^6,7,8^ An unanticipated effect of access to POC-RMPCR was that it led to substantially higher testing rates of residents. Not only was testing 2-fold higher, the increased ratio of confirmed to suspected infections indicated better case detection. By comparison, the case numbers from NHs in the control group were underestimated, given the high percentage positivity among the respiratory virus tests collected. True case numbers likely differed substantially, and the imbalance in case ascertainment contributed to the negative primary outcome.

The reduction in hospital ED transfers was validated in several ways. Increased testing in intervention NHs could have biased the probability of ED transfer for confirmed infection due to increased detection of milder cases. However, the even greater reduction in ED transfers when including cases without a confirmed diagnosis (suspected infections) indicates that the benefit is not fully explained by detection bias. Units in outbreak saw the greatest reduction in ED transfers with the intervention. The difference in outcomes was also accentuated in December and January 2024, when the community incidence of respiratory viruses and risk of outbreak are highest.

Our study findings were consistent with a prior randomized clinical trial of a rapid influenza immunoassay that resulted in 22% fewer transfers to the ED.^14^ Two trials in acute care hospitals that used POC-PCR for influenza only found that faster results led to reduced time spent in isolation precautions and a shorter time to initiation of antiviral therapy.^19,27^ Clark et al^19^ conducted a randomized clinical trial that demonstrated that POC-PCR testing for influenza resulted in a 5-hour faster time to antiviral therapy. Brendish et al^27^ found that more inpatients received antiviral treatment and the length of hospital stay was 1.1 day shorter when POC-RMPCR was used.

Unlike in acute

care hospitals, the additional benefit of POC-RMPCR is the prevention of viral transmission through improved detection and isolation of residents. Time to antiviral therapy for influenza likely played a secondary role on avoided ED transfers. The lack of difference in time to initiation of antiviral therapy for SARS-CoV-2 was possibly related to the use of RATs in control homes. The absence of substantial change in deaths is likely due to low event rates but could be magnified during seasons with a higher case fatality rate or future pandemics.

Mitigation of respiratory outbreaks in NHs has largely focused on pharmacologic interventions, including vaccination and antivirals. A recent scoping review of preventive measures in NHs found comparatively little supporting evidence for many routinely used nonpharmacologic interventions (NPIs).^28^ Our NPI trial emphasized the effect of improved viral respiratory surveillance on resident outcomes. A key lesson for future trials is the need to include severity outcomes to account for detection bias.

The greater than 2-fold increase in testing and detection of confirmed viral respiratory infection represents a major improvement in surveillance among intervention NHs. As a pragmatic trial, testing was left to participating NHs. We encouraged standardized surveillance through weekly IPAC COP meetings, suggesting the improvement was driven by access to POC-RMPCR. We postulated that the accessibility and rapid TAT of the POC-RMPCR enabled a lower threshold for testing residents, especially among those with mild or atypical symptoms.^29^ The rapid TAT and sensitivity likely overcame the downsides of testing NH residents with less severe symptoms, who frequently need to remain in isolation for days while awaiting RMPCR results. Detection of other respiratory viruses was no worse among intervention NHs by selectively forwarding respiratory specimens to reference laboratories for expanded RMPCR.

Cost and increased workload for staff are perceived barriers to adoption of POC-RMPCR in NHs.^30^ Our study addressed concerns about workload given the increased rates of testing by the intervention NHs. While there are fixed upfront costs of POC-RMPCR instruments and ongoing cartridge costs, the direct savings from fewer ED transfers would exceed these costs.^31^ The estimated avoided ED transfers observed in our study translated to approximately 8000 across Canada, 19 000 in the UK, and 64 000 across the US each season. During seasonal hospital surges, these avoided ED transfers could improve health care capacity.^32,33,34^ In a single-payer system, these savings would compensate for the higher cost of using these instruments. Additionally, widespread adoption of POC-RMPCR in NHs would make respiratory virus testing more equitable for residents.

### Strengths and Limitations

Our study had several strengths. First, participating NHs were randomized based on predictors of transmission. Slightly higher crowding index, facility size, and for-profit status favored higher transmission risk in the intervention group, which strengthens our study findings.^35,36^ Second, the study was conducted across a network of NHs in which both groups had similar IPAC and clinical practices by virtue of a shared COP, such that the main difference was testing modality. Third, the effect of community incidence of respiratory viral infections on resident outcomes was minimized given that the trial was completed during the same respiratory season in 1 geographic region. Finally, the pragmatic design in which POC-RMPCR use was left to the discretion of participating NHs provides the clinical effect of this NPI.

There were also study limitations. First, the differential testing between study groups likely biased the primary outcome toward the null. Second, randomization of NHs based on predictors of transmission meant that we could not fully exclude the possibility of resident characteristics driving the differences in resident outcomes. Staff immunization rates are also not available. However, vaccination of residents was similar between groups, and the difference in hospital ED transfers was accentuated during outbreaks. Third, access to SARS-CoV-2 RATs in control NHs may have contributed to a smaller effect size on resident outcomes. Fourth, the trial was conducted during a single viral respiratory season during which the incidence of influenza was greater than the 5-year average.^37^ Finally, as the study was conducted in an urban region, the effect in rural areas where TAT is longer would be even greater.

## Conclusions

This cluster randomized clinical trial found that use of POC-RMPCR instruments in NHs did not change outbreak number or size but decreased the number of ED transfers in the context of increased viral testing, improved case detection, and faster initiation of antiviral therapy for influenza. Seasonal adoption of POC-RMPCR in NHs would mitigate the effect of respiratory outbreaks on resident ED transfers and improve health system capacity.

## Notes

Supplement 1. Trial protocol

Supplement 2. Statistical analysis plan

Supplement 3. eTable 1. A list of the participating nursing homes in the study eTable 2. Confirmed and suspected cases of residents with a respiratory virus infection in intervention and control nursing homes eTable 3. Outbreak characteristics between intervention nursing homes with on-site point of care respiratory multiplex polymerase chain reaction instrument as compared to control nursing homes using standard offsite testing in regional laboratories eTable 4. Outbreak sizes and duration for each allocation group separated by virus eTable 5. The number of nursing home residents with SARS-CoV-2, influenza or respiratory syncytial virus infection and the overall 28-day outcome separated by allocation group eTable 6. The outcomes of residents stratified by virus separated by confirmed respiratory infection versus suspected infection eTable 7. Secondary outcomes between intervention and control nursing homes separated by SARS-CoV-2 and influenza outbreaks eTable 8. Combined transfer and death rates per 100-bed days separated by allocation group, for outbreak and non-outbreak units, compared during the days when nursing home had an ongoing outbreak of SARS-CoV-2, influenza or RSV eTable 9. Proportion of nursing home residents who received antiviral therapy along with median time from symptom onset to antiviral initiation separated by allocation arm and by virus eTable 10. Weekly respiratory multiplex polymerase chain reaction testing volumes for the subgroup of 8 nursing homes in the East region where total testing volumes was counted and separated by allocation group eFigure 1. Overall monthly rate of combined hospital Emergency Department transfer and deaths per 100 beds among all residents in control and intervention nursing homes throughout study period eFigure 2. Time from symptom onset to receipt of antiviral therapy

Supplement 4. Data sharing statement
