# Supplementary material for: Respiratory Outbreak Mitigation With Point-of-Care Testing in Long-Term Care: A Randomized Clinical Trial
Source: JAMA Intern Med. 2026 Jul 6:e262644. Online ahead of print. doi: 10.1001/jamainternmed.2026.2644 (PMC13338844; doi:10.1001/jamainternmed.2026.2644)
Supplement: Supplement 1. — Trial protocol [file jamainternmed-e262644-s001.pdf]

PROTOCOL TITLE:

imProving Respiratory Outbreak Mitigation through Point-of-care Testing in Long Term Care (PROMPT-LTC): A Cluster Randomized Trial

Co-Investigators:

Michael Garron Hospital (MGH): Chris Kandel, Jeff Powis, James Callahan, Brigitte Pascual, Victoria Sepapion.

Humber River Health (HRH): Ian Brasg, Joan Townsend, Beatrise Edelstein

Public Health Ontario (PHO): Kevin Brown

National Microbiology Laboratory (NML): Tracy Taylor, Paul Sundstrom

Sunnybrook Health Science Centre (SHSC): Paul Yip, Rob Kozak, Christina Chan, Jaclyn O'Brien, Kitty Liu, Heather Candon, Jerome Leis

Baycrest Health Sciences Centre: Carla Rosario, Sid Feldman

Long-Term-Care + regional lead: Andrea Moser, Brian Wong

Nurse-led outreach team lead: Swati Rathod, Nicole Apparicio, Kathleen Kirk

Co-Principal Investigators:

Christopher Kandel MD PhD
Division of Infectious Diseases
Michael Garron Hospital
B-508, 825 Coxwell Avenue
Toronto, Ontario, M4C 3E7
Tel: 416-469-6252
email: christopher.kandel@tehn.ca

Jerome A. Leis, MD MSc
Division of Infectious Diseases
Sunnybrook Health Sciences Centre
B103, 2075 Bayview Avenue
Toronto, Ontario M4N 3M5
tel: 416 480-4243
email: jerome.leis@sunnybrook.ca

Clinical Trial.gov Identifier:

PROTOCOL VERSION NUMBER: 1.0

PROTOCOL VERSION DATE: October 9, 2024

Previous Versions:

Table of Contents

1.0 Study Summary 3
2.0 Objectives 4
    2.1 Primary Objectives 4
    2.2 Secondary Objectives 4
    2.3 Hypothesis 4
3.0 Background 4
    3.1 Background 4
4.0 Study Intervention 5
5.0 Procedures Involved 5
    5.1 Study Design 5
    5.2 Data Capture 5
6.0 Inclusion Criteria 6
    6.1 Identification of Eligible LTC homes 6
7.0 Vulnerable Populations 6
8.0 Number of Participating Institutions 6
9.0 Withdrawal of Participants 6
    9.1 Institution Withdrawal 6
    9.2 Waiver of Informed Consent 6
10.0 Risks to Participants 7
11.0 Potential Benefits to Participants 7
12.0 Data Management and Confidentiality 7
    12.1 Sample Size 7
    12.2 Statistical Analysis 8
    12.3 Database 8
    12.4 Data Storage 8
13.0 Provisions to Protect the Privacy Interests of Participants 8
14.0 Setting 9
15.0 Resources Available 9
16.0 Study Sponsor and Ethics 9
17.0 Study Sponsor and Ethics 9
    17.1 Study Timelines 9
    17.2 Knowledge Translation 9
    17.3 Data Sharing 9
18.0 References 10

1.0 Study Summary

**Study Title**	imProving Respiratory Outbreak Mitigation through Point-of-care Testing in Long Term Care (PROMPT-LTC): A Cluster Randomized Trial
**Study Design**	Parallel Group Cluster Randomized Trial
**Main Primary Outcomes**	To determine whether a point-of-care respiratory multiplex platform for respiratory virus testing in long-term care homes can reduce the number and size of respiratory virus outbreaks.
**Research Intervention(s)**	Point-of-Care Respiratory Multiplex Polymerase Chain Reaction Testing Platform
**Study Population**	Long-term care homes supported by Ontario Health Teams
**Sample Size**	24 long-term care homes
**Study Duration**	November 1, 2024 until April 30, 2025
**Study Specific Abbreviations/ Definitions**	COVID-19: coronavirus disease 2019<br>IPAC: Infection Prevention and Control<br>LTCH: Long-Term Care home<br>OHT: Ontario Health Team<br>PHAC: Public Health Agency of Canada<br>POC-RM-PCR: Point-of-Care Respiratory Multiplex Polymerase Chain Reaction<br>RSV: Respiratory Syncytial Virus
**Keywords**	Outbreak mitigation, Rapid PCR testing

2.0 Objectives

2.1 Primary Objectives

To determine whether a POC-RM-PCR platform situated in LTCHs impacts the number of LTCH residents infected with COVID-19, Influenza, or Respiratory Syncytial Virus (RSV)

2.2 Secondary Objectives

The secondary objectives are to determine whether the use of a POC-RM-PCR platform in long-term care homes impacts:

1) The number of COVID-19, Influenza or RSV outbreaks
2) The number of LTCH residents infected during an outbreak of COVID-19, Influenza, or RSV
3) The frequency of hospital transfers for LTCH residents infected with Influenza, COVID-19 or RSV (within 14-days)
4) The number of deaths in LTCH residents infected with Influenza, COVID-19 or RSV (within 28-days)
5) The duration (in days) of outbreaks of Influenza, COVID-19 or RSV as defined by the Public Health Unit
6) The secondary attack rate of high-risk exposures to each LTCH resident with Influenza, COVID-19 or RSV infection

2.3 Hypothesis

The hypothesis of the PROMPT-LTC study is that the deployment of a POC-RM-PCR platform in LTCHs supported by Ontario Health Teams (OHTs) results in a reduction in the number and size of respiratory virus outbreaks as compared to homes that rely on conventional respiratory PCR testing.

3.0 Background

3.1 Background

Outbreaks of seasonal respiratory viruses can propagate rapidly in LTCHs, especially between November and March, resulting in poor resident outcomes. Timely molecular diagnostic testing after identification of a symptomatic resident remains a challenge for COVID and other respiratory viruses due to the logistics of using a reference laboratory. Delays in obtaining results of respiratory virus testing results leads to missed opportunities to implement virus-specific control measures to interrupt transmission (eg. chemoprophylaxis, early isolation) resulting in more secondary cases and prolonged outbreaks, both of which negatively impact LTCH residents.

Use of a POC-RM-PCR testing platform is a potential solution for LTCHs to reduce turnaround time yet whether this can positively impact LTCH resident outcomes remains unknown. Earlier detection of the respiratory virus could lead to faster implementation of outbreak mitigation measures and a shorter time until the start of effective antiviral therapy [1,2]. Use of POC-RM-PCR platforms are now standard practice in some LTCH and increasingly being adopted. Whether to expand the use of POC-RM-PCR platforms across Ontario LTCHs as a standard funded program is an important policy decision that should be informed by evidence given the considerable costs.

A pilot study involving 3 LTCHs and 4 retirement homes in Toronto using Xpert® Xpress CoV-2/Flu/RSV plus (Cepheid) found that this POC-RM-PCR platform resulted in over 36-hour shorter turnaround time with near-perfect performance characteristics (Area Under Curve=0.96), and shorter time to outbreak detection compared to homes who did not have the platform [3]. This pilot study was not designed to determine the impact on resident outcomes.

In partnership with Ontario Health, there is a desire to scale-up use of this platform across LTCHs located within three OHTs during the 2024-2025 respiratory season. This trial aims to assess whether rapid test results for respiratory pathogens are effective in reducing the number and size of respiratory outbreaks.

4.0 Study Intervention

The intervention being evaluated is the use of a POC-RM-PCR platform situated in LTCHs. The Sunnybrook Microbiology and Biochemistry Department, in partnership with point-of-care test experts from the Public Health Agency of Canada (PHAC), will support training of registered LTCH staff and implementation of the POC-RM-PCR instrument in each facility. PHAC has created educational tools to ensure standard operating procedures are followed for using the POC-RM-PCR instruments. Local OHTs including IPAC experts will support LTCHs staff in the use of POC-RM-PCR for syndromic case and contact management including site visits to review results and ensure quality control.

5.0 Procedures Involved

5.1 Study Design

A parallel group cluster randomized trial design will be used to evaluate the impact of a POC-RM-PCR platform in LTCHs in Toronto, Ontario. The study will run from November 1, 2024 until April 30, 2025. All participating LTCHs (Appendix A) supported by an OHT and hospital-based Infection Prevention and Control (IPAC) will be eligible to participate. The three OHTs involved include North Toronto OHT (Sunnybrook Health Sciences Centre), East Toronto Health Partners (Michael Garron Hospital) and North Western OHT (Humber River Health). PCR testing indications, whether through POC-RM-PCR or through conventional means, will be protocolized for syndromic surveillance and high-risk contacts of infected residents to limit outbreak propagation and compared between LTCHs with and without the platform based on current best practice strategies (Appendix B). The availability of POC-RM-PCR will provide results with a faster turnaround time to enable more rapid case management and high-risk contact management (Appendix C). Those LTCHs randomized to continue to receive conventional testing during this study will receive the POC-RM-PCR platform in the following season (November 1, 2025 to April 30, 2026).

5.2 Data Capture

One of the three participating OHTs is integrated within each participating LTCH, ensuring that all new laboratory-confirmed respiratory virus infections are communicated to the OHT at baseline. In turn, each OHT already provides support for resident care and infection control measures to limit outbreak progression. All outcomes as part of this study are routinely collected by IPAC leads in LTCHs along with their local OHT (see Appendix B and D). These outcomes

include results of all respiratory virus tests, the date of symptom onset for a resident with a laboratory confirmed test for influenza, COVID-19 or RSV, the numbers of high-risk contacts for each confirmed case, and the ultimate clinical outcomes. Resident case (confirmed or suspected) outcomes will be categorized into the worst of no hospital transfer, hospital transfer, or death by day 28. Outcomes of high risk contacts will be categorized into no infection, infection, hospitalization, or death.

5.3 Randomization

The unit of randomization will be the LTCH with allocation generated by the study statistician. All participating LTCHs will be ordered by the sum of their scaled bed size and crowding index as both influence the number and size of a respiratory virus outbreak [4]. From this ranked list block randomization of pairs of LTCHs in a 1:1 allocation will be performed to determine those who will receive the POC-RM-PCR platform.

6.0 Inclusion Criteria

6.1 Identification of Eligible LTC homes

Eligible LTCHs for the study will include the 24 that are already engaged with one of the three OHT (Sunnybrook Health Sciences Centre, Michael Garron Hospital or Humber River Health) and have confirmed interest in participating. Each participating LTCH will review the study protocol and procedures and ratify their participation by signing a research agreement with the study sponsor (see Appendix D).

7.0 Vulnerable Populations

As this is an evaluation of the impact of a standard-of-care intervention, there is no possible undue influence or coercion of any potential study participant.

8.0 Number of Participating Institutions

24 LTCHs will participate with 12 LTCHs randomized to receive a POC-RM-PCR platform.

9.0 Withdrawal of Participants

9.1 Institution Withdrawal

Participating LTCHs can withdraw from the study at any time. No further data will be collected following withdrawal, while previously collected data will be retained unless expressly prohibited.

9.2 Waiver of Informed Consent

Given that this study is evaluating an intervention that is standard of care and is restricted to the turnaround time of a respiratory viral test as well as the impracticality of consenting numerous individuals and the very low risk to study participation, a waiver of informed consent for individual participants is being sought. An agreement form will be signed by representatives from each participating LTCH and a study PI (Appendix C).

10.0 Risks to Participants

As this trial is evaluating the impact considered standard of care, there are no risks to study participation. The potential loss of privacy that may arise from data abstraction is minor as the information collected by each OHT is non-identifying and routinely collected as part of the mandate of each OHT. Data will be stored in an encrypted REDCap database housed on secure servers at Michael Garron Hospital. REDCap is a secure, web-based application designed exclusively to support data capture for research studies. Trained members of the LTCH or the respective OHT will be granted access to the REDCap database to input information. Only selected trained study team members will have access to the REDCap database for analysis.

11.0 Potential Benefits to Participants

The potential benefit to the LTCHs who receive the intervention is the earlier provision of results for respiratory virus tests.

12.0 Data Management and Confidentiality

12.1 Sample Size

Sample size estimates for the cluster randomized trial was based on simulation using the mean bed size of LTCHs in Ontario (n=120) that have an average of 4.8 introductions of a respiratory virus per year with 50% progressing to an outbreak (based on the mean number of outbreaks per LTCH in Ontario in 2023-2024 of 2.4). For each outbreak there is an anticipated spread to 11.8% of LTCH residents. The number and size of each outbreak were jointly modeled as binomial counts with overdispersion that varies between 0.8 and 1.2. Using this framework, which was based on publicly available data from Public Health Ontario from 2023-2024, simulations were run to estimate power (Table 1). For a total of 24 LTCHs participating in a cluster trial the expected power is 0.81 with an assumed effect size of 40%, which rises to 96% if the effect size is 50%.

Table 1. Power estimates from simulating a cluster randomized trial with an assumed 40% reduction in the number and size of respiratory virus outbreaks resulting from the use of a point-of-care respiratory multiplex platform for varying numbers of long-term care homes.

Total LTCH Participants	Power
16	0.62
20	0.73
24	0.81
28	0.86
32	0.91

12.2 Statistical Analysis

Summary statistics will be proportions for categorical data and median with interquartile ranges for continuous data. The number of residents in each LTCH with a respiratory virus infection (Influenza, COVID-19, or RSV) will modeled using a negative binomial model adjusted for the scaled bed size and crowding index to estimate the relative risk of the intervention (use of a POC-RM-PCR). The primary analysis will use an Intention-to-Treat design with a sensitivity analysis following a per-protocol design by including only the LTCHs that use the POC-RM-PCR platform more than twice a week on average. Secondary outcomes will also be evaluated using negative binomial models including for the secondary attack rate of identified high-risk contacts, frequency of resident hospital transfers and counts of resident deaths. One subgroup analysis will be performed for the primary outcomes, which will separate the LTCHs into groups based upon the linkage to each individual participating OHT.

12.3 Database

All study personnel will be trained to appropriately handle sensitive personal health information according to the Standard Operating Procedures operationalized at each participating institution. Clinical information will be stored in a REDCap database located on secure servers at Michael Garron Hospital with password protected website access granted to appropriately trained study team members. REDCap is a secure, web-based application designed exclusively to support data capture for research studies. The data system includes password protection and internal quality checks to ensure data are consistent, complete and accurate. Additional benefits of REDCap include (1) 128-bit encryption between the data entry client and the server (https); (2) audit trails for tracking data manipulation and export procedures; (3) automated export procedures for seamless data downloads to common statistical packages; and (4) procedures for importing data from external sources. The REDCap database will be housed at Michael Garron Hospital with access provided to trained study staff overseen by the applicable site investigator or OHT. Quality control of the data will be performed by the study sponsor by review of the information entered into the REDCap database. Queries will be sent to each participating institution to ensure data accuracy.

12.4 Data Storage

All data will be stored for 7 years and then confidentially destroyed. Information entered into the database will be de-identified to the extent possible when exported from REDCap for analysis. All databases will be stored locally for a minimum of 7 years with each participating institution directed by site-specific regulations and procedures.

13.0 Provisions to Protect the Privacy Interests of Participants

If needed, each participant will be assigned a unique study identification number in the master linking log stored in a separate, encrypted electronic file housed on a secure, restricted access server at each participating institution.

14.0 Setting

All LTCHs that partner with the three participating OHTs in Toronto will be eligible to participate. Participation is voluntary and non-participation of LTCHs will have no impact on the working relationship with their local OHT. Those who decide to participate will be asked to

review and sign an agreement template that outlines expectations around use of the POC-RM-PCR equipment and participation in the evaluation. Personnel from each applicable OHT will be responsible for data collection in concert with the LTCH.

15.0 Resources Available

The equipment, cartridges, training and implementation of POC-RM-PCR are being supported by Cepheid, Public Health Agency of Canada, Public Health Ontario, Sunnybrook Health Sciences Centre, Michael Garron Hospital, Humber River Health, and Ontario Health Equipment and Training Fund.

16.0 Study Sponsor and Ethics

Michael Garron Hospital will be the sponsor institution and study approval will be sought from their Research Ethics Board. Each participating LTCH will sign an agreement outlining the expected requirements of study participation, including information transfer to the sponsor.

17.0 Study Sponsor and Ethics

17.1 Study Timelines

LTCHs will be recruited to participate in the study that will run from November 1, 2024 and conclude on April 30, 2025 with final outcomes determined by May 31, 2025. Results will be analyzed and reported by August 30, 2025 with a goal of dissemination in a peer-reviewed manuscript by November, 2025.

17.2 Knowledge Translation

Study results will be published in an open-access peer-reviewed publication and conference abstracts. The study team and partners are well positioned to disseminate study results to policy providers provincially and nationally.

17.3 Data Sharing

Data from this study will be available to all study team members and participating LTCHs. Outside investigators can contact the study team to request access to de-identified data. Requests will be reviewed on a case-by-case basis and will require REB approval.

18.0 References

1. Hammond J, Leister-Tebbe H, Gardner A, et al. Oral Nirmatrelvir for High-Risk, Nonhospitalized Adults with Covid-19. N Engl J Med **2022**;

2. Zhao Y, Gao Y, Guyatt G, et al. Antivirals for post-exposure prophylaxis of influenza: a systematic review and network meta-analysis. Lancet **2024**; 404:764–772.

3. Tan C, Chan CK, Ofner M, et al. Implementation of point-of-care molecular testing for respiratory viruses in congregate living settings. Infect Control Hosp Epidemiol **2024**; :1–5.

4. Brown KA, Jones A, Daneman N, et al. Association Between Nursing Home Crowding and COVID-19 Infection and Mortality in Ontario, Canada. JAMA Intern Med **2021**; 181:229–236.

Appendix A. Long-term care homes (LTCH) that have confirmed interest in implementing POC PCR testing and are committed to receiving support from a local OHT.

Ontario Health Team	LTCH	Bed Size
**Michael Garron Hospital**	1. Midland Gardens Care Community (*Sienna*)	299
	2. Harmony Hills Care Community (*Sienna*)	160
	3. Fountainview Care Community (*Sienna*)*	158
	4. Main Street Terrace (*Extendicare*)	150
	5. True Davidson Acres (*City of Toronto*)	187
	6. Ina Grafton Gage Home (*Responsive*)	128
	7. Chester Village (*Independent*)	203
	8. Nisbet Lodge (*Independent*)	103
	9. Heritage Nursing Home (*Independent*)	201
	10. St. Clair O'Connor Community *(Independent)*	30
	11. Atrium at Kew Beach (MGH ALC site)*	80
**Humber River Health**	12. Downsview LTC	252
	13. Harold & Grace Baker Centre	120
	14. Hawthorne Place Care Centre	269
	15. Norfinch Care Community Nursing Home	160
	16. Ukrainian Care Centre	152
	17. Villa Colombo	391
	18. Village of Humber Heights LTCH	192
	19. West park LTCH	200
	20. Weston Terrace Care Community	224
**Sunnybrook Health Sciences Centre**	21. Baycrest Apotex	472
	22. Isabel and Arthur Meighen Manor	168
	23. Veterans Centre	188
	24. Pine Villa*	68

*Facilities designated as Alternate Level of Care, which encompasses patient populations similar to LTCHs as the vast majority are waiting for placement.

Appendix B. Algorithm for management of new case and contacts of residents with laboratory-confirmed respiratory viral infection (whether conventional or point-of-care PCR used).

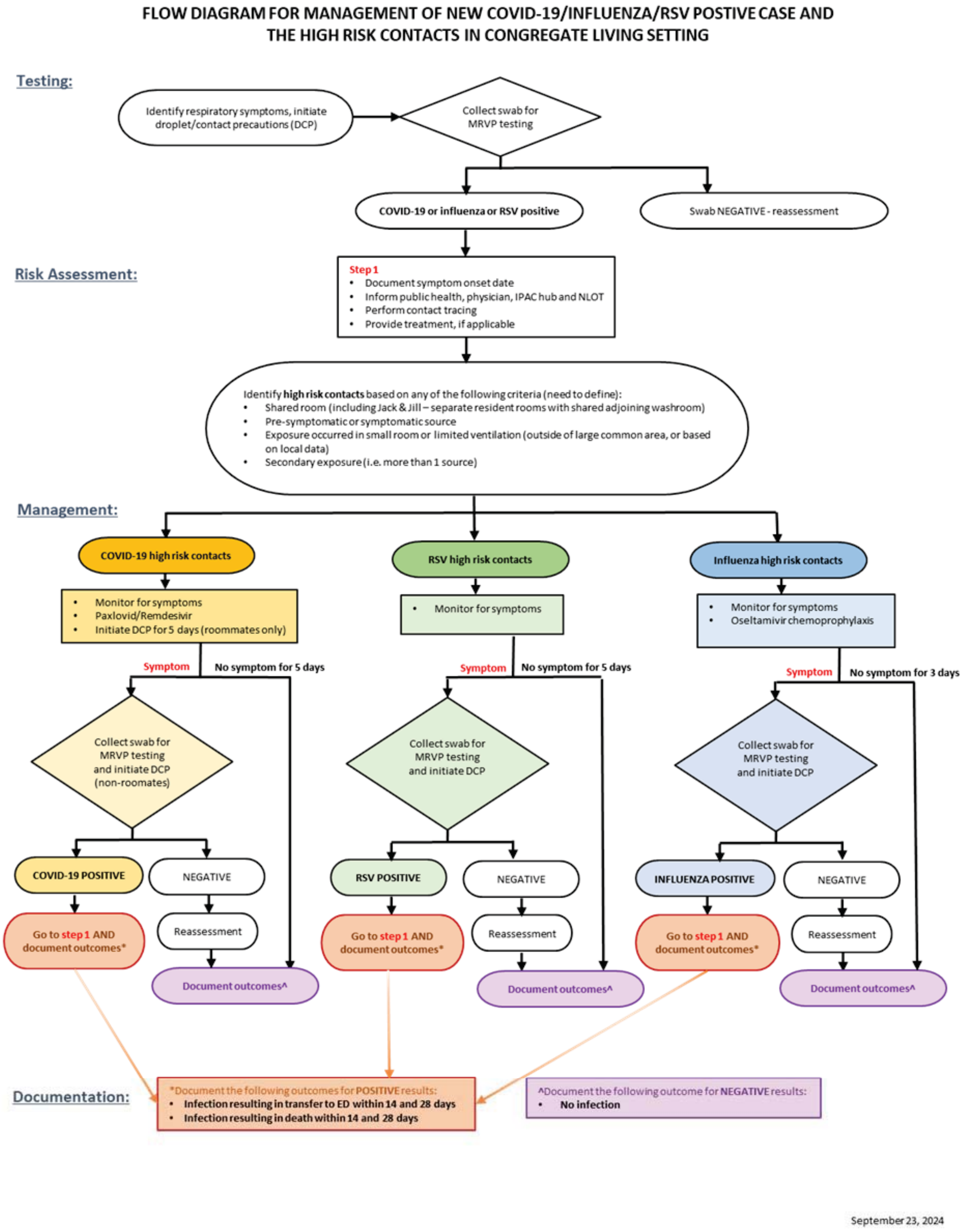

Appendix C. Use of point-of-care testing for early detection, treatment and control of transmission in intervention homes.

Virus	Case management	High Risk Contact Management
SARS CoV-2	Nirmatrelvir/ritonavir OR Remdesivir	Daily testing x 3 days
Influenza	Oseltamivir	Oseltamivir chemoprophylaxis x 7 days
RSV	Inhaled Bronchodilators	Daily testing x 3 days

Appendix D. Research Study statement of agreement to be signed by each long-term care home who wishes to participate in the study.

RESEARCH STUDY - STATEMENT OF AGREEMENT

imProving Respiratory Outbreak Mitigation through Point-of-care Testing in Long Term Care (PROMPT-LTC): A Cluster Randomized Trial

We have reviewed the protocol for this cluster-randomized study in long term care homes (LTCH) and understand that participation is entirely voluntary. The LTCH's decision to participate will have no impact on their relationship with the Ontario Health Teams leading this study (Michael Garron Hospital, Humber River Hospital, Sunnybrook Health Sciences Centre).

By choosing to participate, we understand that the LTCH will:

1. Be randomized to either receive the point-of-care PCR instrument this season (2024-2025) or next season (2025-2026)
2. Use the instrument as directed by local Ontario Health Team
3. Participate in the evaluation in both the 2024-2025 and 2025-2026 respiratory viral season (approximately November 1st to April 30th)
4. Allow the local Ontario Health Team and associated study investigators to record and store securely store non-identifying health information at the sponsor site (Michael Garron Hospital) non-identifying health information about resident outcomes due to Influenza, SARS-CoV-2, Respiratory Syncytial Virus (RSV), and their high-risk contacts, including
   a. Date of onset of symptoms
   b. Time from symptom onset to testing
   c. Results of point-of-care or conventional PCR testing as available
   d. Outcomes related to their infection (no infection, infection, transfer to hospital or death at 14 and 28 days)
5. Agree to allow these aggregate resident outcomes without identifying the LTCH to be shared with Ontario Health and in abstracts or publications.

It is also agreed that at any point throughout the study, the LTCH can decide to withdraw their consent and return the point-of-care instrument.

Given these provisions, we believe that it is reasonable for the following LTCH to participate in this study.

______________________	______________________
Name of LTCH	Ontario Health Team representative
______________________	______________________
Print Name of Signatory	Signature
______________________	______________________
Role/Position at LTCH	Date (dd/mmm/yyyy)

RETURN a signed copy to either Christopher Kandel (christopher.kandel@tehn.ca OR fax at 416-469-6253) or Jerome Leis (jerome.leis@sunnybrook.ca).
