# Supplementary material for: Respiratory Outbreak Mitigation With Point-of-Care Testing in Long-Term Care: A Randomized Clinical Trial
Source: JAMA Intern Med. 2026 Jul 6:e262644. Online ahead of print. doi: 10.1001/jamainternmed.2026.2644 (PMC13338844; doi:10.1001/jamainternmed.2026.2644)
Supplement: Supplement 2. — Statistical analysis plan [file jamainternmed-e262644-s002.pdf]

imProving Respiratory Outbreak Mitigation through Point-of-care Testing in Long Term Care (PROMPT-LTC): A Cluster Randomized Trial

Statistical Analysis Plan
Version 1.0
October 6, 2025

Dr. Christopher Kandel, MD, PhD
Principal Investigator, Infectious Diseases, Michael Garron Hospital, Toronto, Canada

Dr. Jerome A. Leis MD, MSc
Co-Principal Investigator, Infectious Diseases, Sunnybrook Health Sciences Centre, Toronto, Canada

Dr. Kevin A. Brown PhD
Associate Professor, Dalla Lana School of Public Health, Toronto, Canada

Sponsor and Contact addresses

Dr Christopher Kandel, MD, PhD
Michael Garron Hospital
825 Coxwell Avenue, Room J071, Toronto, ON, M4C 3E7
Tel: 416-469-6252; Fax: 416-469-6253
Email: christopher.kandel@tehn.ca

TECHNICAL APPROVERS

Chris Kandel

Dr. C. Kandel, MD, PhD Principal Investigator

October 6, 2025

Date of Approval

Dr. J. Leis, MD, MSc co-Principal Investigator

Oct 6, 2025

Date of Approval

Dr. K. Brown, PhD Senior Biostatistician

October 6, 2025

Date of Approval

Table of Contents

Sponsor and Contact addresses 1
Abbreviations 4
Related Papers 5
1.0 Introduction 6
___1.1 Study Hypothesis 6
___1.2 Primary Objective 6
___1.3 Secondary Objectives 6
2.0 Study Design 7
3.0 Sequence of Planned Analysis 7
4.0 Sample Size Considerations 7
5.0 Randomization 7
6.0 Analysis Populations 8
___6.1 General Consideration for Data Analysis 8
___6.2 Outbreak definitions and Endpoints 8
7.0 Baseline Comparison 9
8.0 Efficacy Analyses 9
___8.1 Primary outcome analysis 9
___8.2 Secondary outcome analysis 9
___8.3 Subgroup Analyses 10
___8.4 Exploratory Analysis 10
9.0 Reporting Conventions 11

Abbreviations

COVID-19:	Coronavirus disease 2019
IPAC:	Infection Prevention and Control
LTCH:	Long-Term Care home
OHT:	Ontario Health Team
PHAC:	Public Health Agency of Canada
POC-RM-PCR	Point-of-Care Respiratory Multiplex Polymerase Chain Reaction
RSV:	Respiratory Syncytial Virus

Related Papers

- Protocol PROMPT-LTC Version 1.1 May 12, 2025
    - imProving Respiratory Outbreak Mitigation through Point-of-care Testing in Long Term Care (PROMPT-LTC): A Cluster Randomized Trial

- Database Entry general: REDCap

- GCP/ICH guidelines:
    - https://ichgcp.net/8-essential-documents-for-the-conduct-of-a-clinical-trial
    - https://ichgcp.net/2-the-principles-of-ich-gcp-2

1.0 Introduction

Outbreaks of seasonal respiratory viruses can propagate rapidly in long-term care homes (LTCHs), especially between November and March, resulting in poor resident outcomes. Timely molecular diagnostic testing after identification of a symptomatic resident remains a challenge for COVID-19 and other respiratory viruses due to the logistics of using a reference laboratory. Delays in obtaining results of respiratory virus testing results leads to missed opportunities to implement virus-specific antiviral treatment and/or local control measures to interrupt transmission (eg. chemoprophylaxis, early isolation) resulting in more secondary cases and severe resident outcomes.

Use of a Point-of-Care Respiratory Multiplex Polymerase Chain Reaction (POC-RM-PCR) testing platform is a potential solution for LTCHs to reduce turnaround time yet whether this can positively impact LTCH resident outcomes remains unknown. Earlier detection of the respiratory virus could lead to faster implementation of outbreak mitigation measures and a shorter time until the start of effective antiviral therapy. Use of POC-RM-PCR platforms are increasingly being adopted across LTCHs but their impact on resident outcomes is not known. Whether to expand the use of POC-RM-PCR platforms across Ontario LTCHs as a standard funded program is an important policy decision that should be informed by evidence given the upfront costs.

1.1 Study Hypothesis

The hypothesis of the PROMPT-LTC study is that the deployment of a POC-RM-PCR platform in LTCHs supported by Ontario Health Teams (OHTs) results in a reduction in the number and size of respiratory virus outbreaks and associated severe outcomes (transfer to hospital or death) as compared to homes that rely on conventional respiratory PCR testing.

1.2 Primary Objective

To determine whether a POC-RM-PCR platform situated in LTCHs impacts the number of LTCH residents infected with COVID-19, Influenza, or Respiratory Syncytial Virus (RSV)

1.3 Secondary Objectives

1) The number of COVID-19, Influenza or RSV outbreaks
2) The number of LTCH residents infected during an outbreak of COVID-19, Influenza, or RSV
3) The frequency of hospital transfers for LTCH residents infected with Influenza, COVID-19 or RSV (within 14-days)
4) The number of deaths in LTCH residents infected with Influenza, COVID-19 or RSV (within 28-days)
5) The duration (in days) of outbreaks of Influenza, COVID-19 or RSV as defined by the Public Health Unit

6) The secondary attack rate of high-risk exposures to each LTCH resident with Influenza, COVID-19 or RSV infection

2.0 Study Design

A parallel group cluster randomized trial design will be used to evaluate the impact of a POC-RM-PCR platform in LTCHs in Toronto, Ontario. The study will run from November 1, 2024 until April 30, 2025. All participating LTCHs supported by an OHT and hospital-based Infection Prevention and Control (IPAC) will be eligible to participate. The three OHTs involved include North Toronto OHT (Sunnybrook Health Sciences Centre), East Toronto Health Partners (Michael Garron Hospital) and North Western OHT (Humber River Health). PCR testing indications, whether through POC-RM-PCR or through conventional means, is already protocolized for syndromic surveillance and high-risk contacts of infected residents to limit outbreak propagation and compared between LTCHs with and without the platform based on current best practice strategies.

3.0 Sequence of Planned Analysis

The final CRF-data, checked for plausibility and validity, will be transferred to the biostatisticians. All derived and transformed data will be computed via a syntax/program-file (file containing all commands of data handling and analysis). Only after this, the final statistical analysis, as detailed in this plan, will be performed. All statistical analysis will be performed via syntax/program files.

4.0 Sample Size Considerations

Sample size estimates for the cluster randomized trial was based on simulation using the mean bed size of LTCHs in Ontario (n=120) that have an average of 4.8 introductions of a respiratory virus per year with 50% progressing to an outbreak (based on the mean number of outbreaks per LTCH in Ontario in 2023-2024 of 2.4). For each outbreak there is an anticipated spread to 11.8% of LTCH residents. The number and size of each outbreak were jointly modeled as binomial counts with overdispersion that varies between 0.8 and 1.2 standard deviations. Using this framework, which was based on publicly available data from Public Health Ontario from 2023-2024, simulations were run to estimate power. For a total of 24 LTCHs participating in a cluster trial the expected power is 0.81 with an assumed effect size of 40%, which rises to 96% if the effect size is 50%.

5.0 Randomization

The unit of randomization will be the LTCH with allocation generated by the study statistician. All participating LTCHs will be ordered by the sum of their scaled bed size and crowding index as both influence the number and size of a respiratory virus outbreak. From this ranked list block randomization of pairs of LTCHs in a 1:1 allocation will be performed to determine those who will receive the POC-RM-PCR platform.

6.0 Analysis Populations

All units from each participating long-term care are included in the intention-to-treat analysis.

6.1 General Consideration for Data Analysis

All analysis will be performed in R version 4.4.3. All data handling and analysis must be saved in a syntax/program file. Any analysis requiring significance testing will use a two-sided test at the 0.05 significance level. Confidence interval of 95% will be calculated.

6.2 Outbreak definitions and Endpoints

Outbreak Definition

Outbreak definition: Two or more cases with a confirmed viral infection on the same unit within a 14-day period. An outbreak is declared over when no further transmission has occurred over 2 incubation periods. The start date of the outbreak is the date of the first confirmed case, and the end date is the last confirmed case. Viral outbreaks that occur concurrently on a unit will be separated into individual viruses with their own start and stop date based on the definition above. Suspected cases include transfers and deaths that occur during a laboratory-confirmed respiratory outbreak and up to 14-days after the outbreak is declared over. If a suspected case cannot be attributed to an outbreak (for example when more than one virus is circulating in a unit simultaneously) then it will be omitted as a case.

Primary Endpoint: The primary endpoint is the outbreak number and number of secondary cases of each outbreak.

Secondary Endpoints

1) The number of COVID-19, Influenza or RSV outbreaks
2) The number of LTCH residents infected during an outbreak of COVID-19, Influenza, or RSV
3) The frequency of hospital transfers for LTCH residents infected with Influenza, COVID-19 or RSV (within 14-days)
4) The number of deaths in LTCH residents infected with Influenza, COVID-19 or RSV (within 28-days)
5) The duration (in days) of outbreaks of Influenza, COVID-19 or RSV as defined by the Public Health Unit
6) The secondary attack rate of high-risk exposures to each LTCH resident with Influenza, COVID-19 or RSV infection

7.0 Baseline Comparison

The following variables will be described for each group (standard of care and POC-RM-PCR): number of units per home, number of beds per unit, crowding index, proportion that are for-profit, location and proportion with a current RSV, Influenza or COVID-19 vaccine. The number and percentage will be displayed for each binary or categorical variable and median with interquartile range for continuous variables. No comparisons between the groups will be presented.

8.0 Efficacy Analyses

The analysis will be performed on the Intention to Treat population.

8.1 Primary outcome analysis: The primary outcome is the relative risk of outbreak-associated cases, estimated using a joint model of the outbreak number and size. Outbreak number will be modeled using a random effects Poisson model with virus and allocation included as a covariates. Outbreak size will be modeled using a random effects binomial count model with the number of secondary cases and non-cases in each outbreak and with virus and allocation included as co-variates. The effect size coefficients for allocation for the outbreak and outbreak size models will be summed in a joint model using the meta-analysis package metafor version 4.8-0.

The primary outcome will be presented as a pooled estimate of the joint distribution of the model with a 95% confidence interval a P-value. Larger values are indicative of a worse outcome.

8.2 Secondary outcome analysis

8.2.1 – The number of COVID-19, Influenza or RSV outbreaks

The counts of the number of outbreaks will be presented for each group and separately by virus. A Poisson model will be fit to determine the absolute differences between groups and the incidence rate ratio.

8.2.2 – The number of LTCH residents infected during an outbreak of COVID-19, Influenza, or RSV

The counts of the number of initial and secondary cases will be presented for each group and separated by each virus individually. Cases will be broken down into confirmed (tested positive for the applicable prevailing virus during an outbreak or suspected, which is defined as a hospital transfer or death of a resident on the outbreak unit irrespective of positive viral test). The proportion of secondary cases for each outbreak will be assessed with a binomial model and then compared for Influenza and COVID-19 separately. The absolute proportion of susceptible residents who are a case during the outbreak will be presented along the absolute difference and odds ratio.

8.2.3 – The frequency of hospital transfers for LTCH residents infected with Influenza, COVID-19 or RSV (within 14-days)

Counts and percentages from each group will be presented in total and for Influenza, COVID-19, and RSV separately. The probability of a hospital transfer within 14 days of symptom onset will be modeled using logistic regression with absolute differences and an odds ratio presented.

8.2.4 – The number of deaths in LTCH residents infected with Influenza, COVID-19 or RSV (within 28-days)

Counts and percentages from each group will be presented in total and for Influenza, COVID-19 and RSV separately. The probability of a death (in home or after a hospital transfer) within 28 days of symptom onset will be modeled using logistic regression with absolute differences and an odds ratio presented.

8.2.5 – The duration (in days) of outbreaks of Influenza, COVID-19 or RSV as defined by the Public Health Unit

If available, the outbreak duration in days will be presented with the median and interquartile range in total and for each virus separately.

8.2.6 – The secondary attack rate of high-risk exposures to each LTCH resident with Influenza, COVID-19 or RSV infection

The probability of a secondary transmission among identified high-risk contact of a case during an outbreak will be modeled. The absolute differences and odds ratio will be reported.

8.3 Subgroup Analyses

Each of the analyses above will be performed for Influenza and COVID-19 outbreaks separately.

8.4 Exploratory Analysis

8.3.1 – The number of tests performed

The number of positive and negative tests performed at the 8 facilities in the East region (the only facilities that captured all test results) will be presented as the mean number of weekly number of tests performed and compared between groups.

8.3.2. – Test positivity

The proportion of positive tests among the total number of tests performed will be presented as the overall proportion of tests and compared between groups.

8.3.3 – Overall monthly deaths and transfers

The monthly counts of hospital transfers and deaths will be presented for each group and compared.

8.3.4 – Transfer and death rates on outbreak and non-outbreak units

The transfer and death rates on each unit in an outbreak will be compared to the rates of transfers and deaths on non-outbreak units over the same outbreak period will be presented as a rate per 100 resident days. Outbreaks occurring simultaneously will be removed and a secondary analysis will include only periods where one outbreak in a home was occurring.

8.3.5 – Time from symptom onset to treatment

The time (in days) from symptom onset to treatment will be presented for residents with COVID-19 or influenza for all residents and each virus separately. The time in days will be compared between allocation groups.

9.0 Reporting Conventions

The ICH-GCP guideline: "Structure and content of clinical study reports" (link: https://www.ema.europa.eu/en/documents/scientific-guideline/ich-e-3-structure-content-clinical-study-reports-step-5_en.pdf) and the CONSORT 2010 Statement (link: http://www.consort-statement.org/consort-2010) , for reporting will be followed.

According to this foundation the contents of the final report are:

- Copy of final complete general database
- Tables of results: to be completed when database is ready.
- Statistical report: to be completed when analysis is ready.
- Manuscript: to be completed when analysis is ready.
