# Supplementary material for: Respiratory Outbreak Mitigation With Point-of-Care Testing in Long-Term Care: A Randomized Clinical Trial
Source: JAMA Intern Med. 2026 Jul 6:e262644. Online ahead of print. doi: 10.1001/jamainternmed.2026.2644 (PMC13338844; doi:10.1001/jamainternmed.2026.2644)
Supplement: Supplement 3. — eTable 1. A list of the participating nursing homes in the study eTable 2. Confirmed and suspected cases of residents with a respiratory virus infection in intervention and control nursing homes eTable 3. Outbreak characteristics between intervention nursing homes with on-site point of care respiratory multiplex polymerase chain reaction instrument as compared to control nursing homes using standard offsite testing in regional laboratories eTable 4. Outbreak sizes and duration for each allocation group separated by virus eTable 5. The number of nursing home residents with SARS-CoV-2, influenza or respiratory syncytial virus infection and the overall 28-day outcome separated by allocation group eTable 6. The outcomes of residents stratified by virus separated by confirmed respiratory infection versus suspected infection eTable 7. Secondary outcomes between intervention and control nursing homes separated by SARS-CoV-2 and influenza outbreaks eTable 8. Combined transfer and death rates per 100-bed days separated by allocation group, for outbreak and non-outbreak units, compared during the days when nursing home had an ongoing outbreak of SARS-CoV-2, influenza or RSV eTable 9. Proportion of nursing home residents who received antiviral therapy along with median time from symptom onset to antiviral initiation separated by allocation arm and by virus eTable 10. Weekly respiratory multiplex polymerase chain reaction testing volumes for the subgroup of 8 nursing homes in the East region where total testing volumes was counted and separated by allocation group eFigure 1. Overall monthly rate of combined hospital Emergency Department transfer and deaths per 100 beds among all residents in control and intervention nursing homes throughout study period eFigure 2. Time from symptom onset to receipt of antiviral therapy [file jamainternmed-e262644-s003.pdf]

Supplemental Online Content

Kandel C, Oriotis D, Candon HL, et al. Respiratory outbreak mitigation with point-of-care testing in long-term care: a randomized clinical trial. *JAMA Intern Med*. Published online July 6, 2026. doi:10.1001/jamainternmed.2026.2644

eTable 1. A list of the participating nursing homes in the study
eTable 2. Confirmed and suspected cases of residents with a respiratory virus infection in intervention and control nursing homes
eTable 3. Outbreak characteristics between intervention nursing homes with on-site point of care respiratory multiplex polymerase chain reaction instrument as compared to control nursing homes using standard offsite testing in regional laboratories
eTable 4. Outbreak sizes and duration for each allocation group separated by virus
eTable 5. The number of nursing home residents with SARS-CoV-2, influenza or respiratory syncytial virus infection and the overall 28-day outcome separated by allocation group
eTable 6. The outcomes of residents stratified by virus separated by confirmed respiratory infection versus suspected infection
eTable 7. Secondary outcomes between intervention and control nursing homes separated by SARS-CoV-2 and influenza outbreaks
eTable 8. Combined transfer and death rates per 100-bed days separated by allocation group, for outbreak and non-outbreak units, compared during the days when nursing home had an ongoing outbreak of SARS-CoV-2, influenza or RSV
eTable 9. Proportion of nursing home residents who received antiviral therapy along with median time from symptom onset to antiviral initiation separated by allocation arm and by virus
eTable 10. Weekly respiratory multiplex polymerase chain reaction testing volumes for the subgroup of 8 nursing homes in the East region where total testing volumes was counted and separated by allocation group
eFigure 1. Overall monthly rate of combined hospital Emergency Department transfer and deaths per 100 beds among all residents in control and intervention nursing homes throughout study period
eFigure 2. Time from symptom onset to receipt of antiviral therapy

This supplemental material has been provided by the authors to give readers additional information about their work.

© 2026 Kandel C et al. *JAMA Internal Medicine*.

eTable 1

A list of the participating nursing homes in the study.

**Ontario Health Team**	**Long-term Care Home**
**East**	Harmony Hills Care Community<br>Fountainview Care Community<br>Main Street Terrace<br>Ina Grafton Gage Home<br>Chester Village<br>Nisbet Lodge<br>Heritage Nursing Home<br>Atrium at Kew Beach
**Northwest**	Downsview Long-Term Care Centre<br>Harold & Grace Baker Centre<br>Hawthorne Place Care Centre<br>Humber Meadows<br>Ukrainian Care Centre<br>Villa Colombo<br>West Park Long-Term Care Centre<br>Weston Terrace Care Community
**North**	Baycrest Apotex<br>Isabel and Arthur Meighen Manor<br>Veterans Centre<br>Pine Villa

© 2026 Kandel C et al. *JAMA Internal Medicine*.

eTable 2

Confirmed and suspected cases of residents with a respiratory virus infection in intervention and control nursing homes. Confirmed cases includes residents with a positive viral test for SARS-CoV-2, influenza or respiratory syncytial virus. Suspected cases include residents of an outbreak unit who did not have a positive test but died or were transferred to a hospital Emergency Department between the start of an outbreak and up to 14 days from the last confirmed outbreak case.

**Cases per 100 beds**	**Overall**	**Control**	**Intervention**
Confirmed viral respiratory infections			
Overall	17.5	17.2	17.8
SARS-CoV-2	10.2	8.3	11.6
Influenza	5.5	6.8	4.6
RSV	1.8	2.1	1.6
Suspected viral respiratory infections			
Overall	6.1	8.5	4.2
SARS-CoV-2	2.8	3.7	2.0
Influenza	2.5	3.2	2.0
RSV	0.8	1.6	0.3

© 2026 Kandel C et al. *JAMA Internal Medicine*.

eTable 3

Outbreak characteristics between intervention nursing homes with an on-site point of care respiratory multiplex polymerase chain reaction instrument as compared to control nursing homes using standard offsite testing in regional laboratories.

**Measures**[1]	**Overall**	**Control** (n=51)	**Intervention** (n=62)
Virus			
SARS-CoV-2	55 / 113 (49%)	21 / 51 (41%)	34 / 62 (55%)
Influenza	44 / 113 (39%)	21 / 51 (41%)	23 / 62 (37%)
RSV	14 / 113 (12%)	9 / 51 (18%)	5 / 62 (8.1%)
Confirmed viral respiratory infection			
Total Outbreak Size	4 (2, 7)	3 (2, 6)	4 (2, 7)
Initial Cases Per Outbreak	1 (1, 2)	1 (1, 1)	1 (1, 2)
Secondary Cases Per Outbreak	2 (1, 5)	2 (1, 5)	3 (1, 6)
Confirmed and suspected viral respiratory infection			
Total Outbreak Size	7 (4, 10)	7 (4, 11)	7 (4, 9)
Initial Cases Per Outbreak	1 (1, 2)	1 (1, 2)	1 (1, 2)
Secondary Cases Per Outbreak	5 (2, 8)	6 (2, 9)	5 (2, 8)
Outbreak Length (days from first to last confirmed case)	6 (3, 10)	7 (4, 11)	5 (2, 10)

[1]Unless specified, n / N (%) or Median (Q1, Q3).

© 2026 Kandel C et al. *JAMA Internal Medicine*.

eTable 4

Outbreak sizes and duration for each allocation group separated by virus (SARS-CoV-2, influenza and respiratory syncytial virus).

	SARS-CoV-2		Influenza		RSV	
**Characteristic**	**Control** N = 21[1]	**Intervention** N = 34[1]	**Control** N = 21[1]	**Intervention** N = 23[1]	**Control** N = 9[1]	**Intervention** N = 5[1]
Confirmed viral respiratory infection						
Total Outbreak Size	5 (3, 7)	6 (3, 8)	3 (2, 6)	3 (2, 6)	2 (2, 3)	3 (2, 4)
Initial Cases Per Outbreak	1 (1, 1)	1 (1, 2)	1 (1, 2)	1 (1, 2)	1 (1, 1)	1 (1, 2)
Secondary Cases Per Outbreak	3 (2, 6)	3 (2, 7)	2 (1, 5)	1 (1, 4)	1 (1, 2)	2 (1, 3)
Confirmed and suspected viral respiratory infection						
Total Outbreak Size	8 (6, 12)	7 (4, 9)	7 (2, 11)	5 (2, 8)	4 (3, 5)	4 (3, 7)
Initial Cases Per Outbreak	1 (1, 1)	1 (1, 2)	1 (1, 2)	1 (1, 2)	1 (1, 1)	1 (1, 2)
Secondary Cases Outbreak Size	7 (5, 9)	6 (3, 8)	6 (1, 9)	3 (1, 7)	3 (2, 4)	3 (2, 6)
Outbreak Length (days from first to last case)	8 (4, 11)	7 (3, 12)	4 (3, 7)	4 (1, 10)	9 (7, 11)	3 (1, 8)

[1]Median (Q1, Q3)

© 2026 Kandel C et al. *JAMA Internal Medicine*.

eTable 5

The number of nursing home residents with SARS-CoV-2, influenza or respiratory syncytial virus infection and the overall 28-day outcome separated by allocation group. Suspected cases include residents of an outbreak unit who did not have a positive test but died or were transferred to a hospital Emergency Department between the start of an outbreak and up to 14 days from the last confirmed outbreak case.

**Characteristic**	**Overall**<br>N = 937[1]	**Control**<br>N = 444[1]	**Intervention**<br>N = 493[1]
Virus			
SARS-CoV-2	513 / 937 (55%)	208 / 444 (47%)	305 / 493 (62%)
Influenza	319 / 937 (34%)	173 / 444 (39%)	146 / 493 (30%)
RSV	105 / 937 (11%)	63 / 444 (14%)	42 / 493 (8.5%)
Confirmed viral respiratory infection outcomes			
Recovered	593 / 937 (63%)	250 / 444 (56%)	343 / 493 (70%)
Transfer to hospital	72 / 937 (7.7%)	38 / 444 (8.6%)	34 / 493 (6.9%)
Transfer and deceased	13 / 937 (1.4%)	4 / 444 (0.9%)	9 / 493 (1.8%)
Deceased within facility	17 / 937 (1.8%)	5 / 444 (1.1%)	12 / 493 (2.4%)
Suspected viral respiratory infection outcomes			
Transfer to hospital	192 / 937 (20%)	121 / 444 (27%)	71 / 493 (14%)
Transfer and deceased	22 / 937 (2.3%)	14 / 444 (3.2%)	8 / 493 (1.6%)
Deceased within facility	28 / 937 (3.0%)	12 / 444 (2.7%)	16 / 493 (3.2%)

[1] n / N (%)

© 2026 Kandel C et al. *JAMA Internal Medicine*.

eTable 6

The outcomes of residents stratified by virus separated by confirmed respiratory infection versus suspected infection. Suspected infection included residents of an outbreak unit who did not have a positive test but died or were transferred to a hospital Emergency Department between the start of an outbreak and up to 14 days from the last confirmed outbreak case.

	SARS-CoV-2		Influenza		RSV	
**Outcome**	**Control** N = 208[1]	**Intervention** N = 305[1]	**Control** N = 173[1]	**Intervention** N = 146[1]	**Control** N = 63[1]	**Intervention** N = 42[1]
Confirmed viral respiratory infection						
Recovered	126 / 208 (61%)	240 / 305 (79%)	93 / 173 (54%)	73 / 146 (50%)	31 / 63 (49%)	30 / 42 (71%)
Transfer to hospital	13 / 208 (6.3%)	14 / 305 (4.6%)	20 / 173 (12%)	16 / 146 (11%)	5 / 63 (7.9%)	4 / 42 (9.5%)
Transfer and deceased	3 / 208 (1.4%)	2 / 305 (0.7%)	1 / 173 (0.6%)	6 / 146 (4.1%)	0 / 63 (0%)	1 / 42 (2.4%)
Deceased within facility	2 / 208 (1.0%)	4 / 305 (1.3%)	3 / 173 (1.7%)	7 / 146 (4.8%)	0 / 63 (0%)	1 / 42 (2.4%)
Suspected viral respiratory infection						
Transfer to hospital	55 / 208 (26%)	33 / 305 (11%)	44 / 173 (25%)	33 / 146 (23%)	22 / 63 (35%)	5 / 42 (12%)
Transfer and deceased	4 / 208 (1.9%)	4 / 305 (1.3%)	7 / 173 (4.0%)	3 / 146 (2.1%)	3 / 63 (4.8%)	1 / 42 (2.4%)
Deceased within facility	5 / 208 (2.4%)	8 / 305 (2.6%)	5 / 173 (2.9%)	8 / 146 (5.5%)	2 / 63 (3.2%)	0 / 42 (0%)

[1] n / N (%)

© 2026 Kandel C et al. *JAMA Internal Medicine*.

eTable 7

Secondary outcomes between intervention and control nursing homes separated by SARS-CoV-2 and influenza outbreaks. These outcomes include confirmed and suspected cases combined.

Outcome	Control	Intervention	Absolute Difference (Intervention – Control, 95% CI)	Odds Ratio (95% CI)	p-value
SARS-CoV-2					
Outbreaks	0.34 (0.14, 0.53)	0.42 (0.24, 0.60)	0.09 (-0.15, 0.33)	1.27 (0.65, 2.46)*	0.484
Outbreak Size	6.25 (5.48, 7.08)	5.69 (5.26, 6.17)	-0.57 (-1.47, 0.32)	1.18 (0.76, 1.81)	0.458
Death	0.06 (-0.00, 0.13)	0.06 (0.00, 0.11)	-0.02 (-0.18, 0.14)	0.87 (0.23, 3.35)	0.841
Transfer	0.35 (0.23, 0.46)	0.15 (0.10, 0.21)	-0.20 (-0.32, -0.07)	0.33 (0.17, 0.64)	**<0.001**
Proportion of Secondary Cases per Outbreak	0.84 (0.77, 0.90)	0.78 (0.72, 0.85)	-0.05 (-0.14, 0.04)	0.71 (0.38, 1.34)	0.295
Influenza					
Outbreaks	0.32 (0.32, 0.32)	0.29 (0.29, 0.30)	-0.03 (-0.03, -0.03)	0.91 (0.90, 0.92)*	**<0.001**
Outbreak Size	5.39 (4.72, 6.10)	3.51 (3.25, 3.81)	-1.88 (-2.61, -1.15)	0.79 (0.41, 1.54)	0.492
Death	0.09 (0.02, 0.16)	0.16 (0.07, 0.26)	0.08 (-0.04, 0.19)	1.98 (0.69, 5.66)	0.202
Transfer	0.38 (0.26, 0.50)	0.35 (0.24, 0.46)	-0.03 (-0.17, 0.12)	0.88 (0.44, 1.74)	0.713
Proportion of Secondary Cases per Outbreak	0.77 (0.68, 0.85)	0.75 (0.66, 0.83)	-0.02 (-0.15, 0.11)	0.89 (0.46, 1.71)	0.720

*Incidence Rate Ratio instead of Odds Ratio

© 2026 Kandel C et al. *JAMA Internal Medicine*.

eTable 8

Combined transfer and death rates per 100-bed days separated by allocation group, for outbreak and non-outbreak units, compared during the days when nursing home had an ongoing outbreak of SARS-CoV-2, influenza or RSV. Rates are shown when a single outbreak was ongoing and when multiple outbreaks were occurring simultaneously.

	Group	Outbreak Rate (per 100 bed-days)	Non-Outbreak Rate (per 100 bed-days)
One Outbreak Ongoing	Control	1.34	0.20
	Intervention	0.50	0.13
Multiple Outbreaks Ongoing	Control	1.34	0.32
	Intervention	0.50	0.16

© 2026 Kandel C et al. *JAMA Internal Medicine*.

eTable 9

Proportion of nursing home residents who received antiviral therapy along with median (interquartile range) time from symptom onset to antiviral initiation separated by allocation arm and by virus (SARS-CoV-2 and influenza infections only given approved antiviral therapy is available).

	Characteristic	Overall	Control	Intervention
Overall	Received Treatment	426 / 695 (61%)	179 / 297 (60%)	247 / 398 (62%)
	Time to Treatment (days)	1.0 (0.0, 2.0)	2.0 (1.0, 4.0)	1.0 (0.0, 2.0)
SARS-CoV-2	Received Treatment	240 / 404 (59%)	80 / 144 (56%)	160 / 260 (62%)
	Time to Treatment (days)	1.0 (1.0, 2.0)	1.0 (0.0, 2.0)	1.0 (1.0, 2.0)
Influenza	Received Treatment	185 / 219 (84%)	98 / 117 (84%)	87 / 102 (85%)
	Time to Treatment (days)	1.0 (0.0, 3.0)	3.0 (1.0, 4.0)	0.0 (0.0, 1.0)

© 2026 Kandel C et al. *JAMA Internal Medicine*.

eTable 10

Weekly respiratory virus multiplex polymerase chain reaction testing volumes for the subgroup of 8 nursing homes in the East region where total testing volumes was counted (all positive and negative tests) and separated by allocation group.

Home	Allocation	Avg. Tests/Week	Avg. Tests/Week/Resident	Percent Positivity
1	Intervention	2.44	0.030	39
2	Control	4.56	0.027	76
3	Control	0.56	0.004	50
4	Intervention	2.88	0.018	49
5	Intervention	1.24	0.010	48
6	Control	0.56	0.006	57
7	Control	1.24	0.012	87
8	Intervention	8.20	0.044	35
	Control (Overall)	1.73	0.012	75
	Intervention (Overall)	3.69	0.026	39

© 2026 Kandel C et al. *JAMA Internal Medicine*.

eFigure 1

Overall monthly rate of combined hospital Emergency Department transfer and deaths per 100 beds among all residents in control and intervention nursing homes throughout study period, inclusive of non-outbreak, suspected viral respiratory infection and confirmed cases respiratory viral infection. Confirmed cases includes residents with a positive viral test for SARS-CoV-2, influenza or respiratory syncytial virus. Suspected cases include residents of an outbreak unit who did not have a positive viral test but died or were transferred to a hospital Emergency Department between the start of an outbreak and up to 14 days from the last confirmed outbreak case.

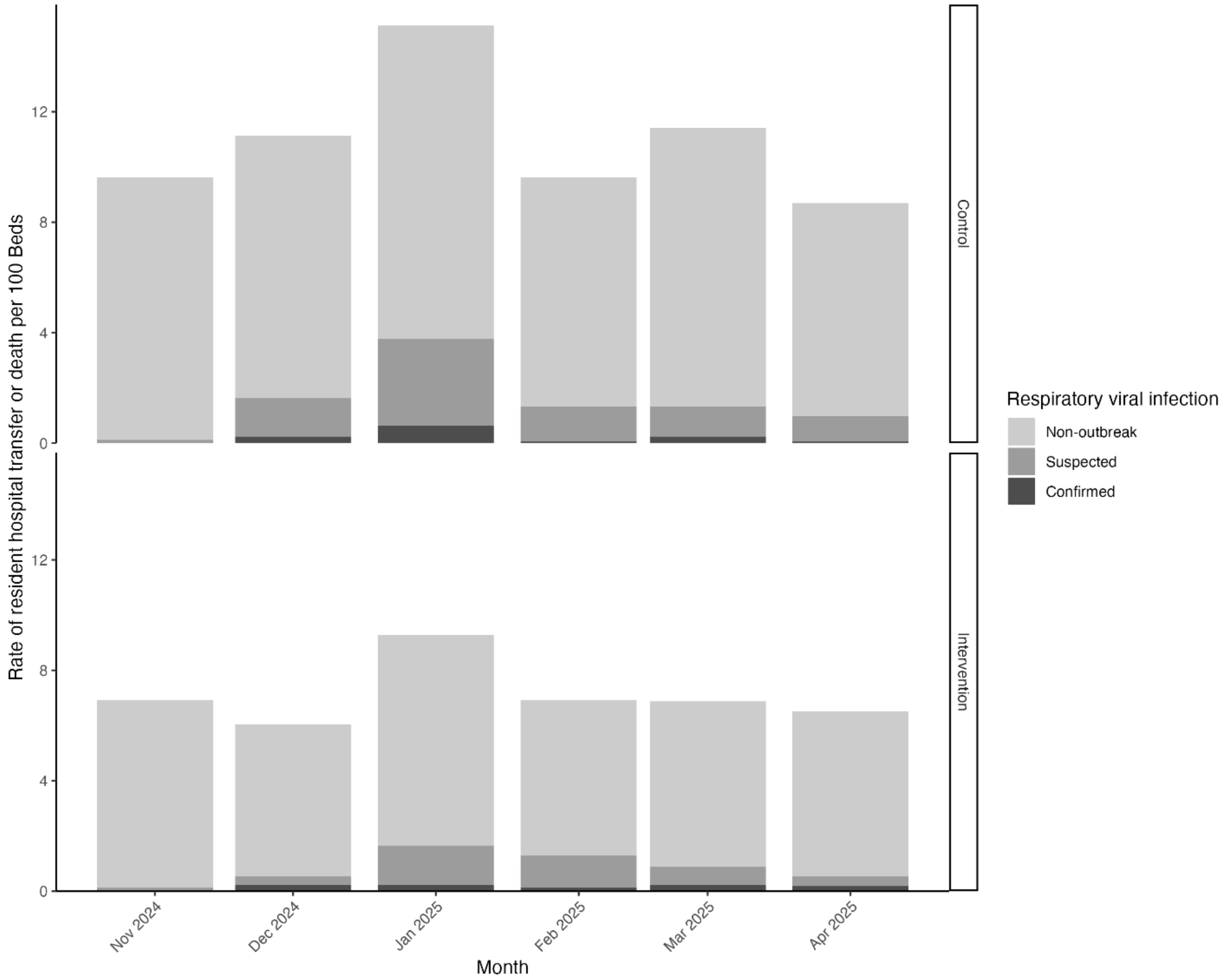

© 2026 Kandel C et al. *JAMA Internal Medicine*.

eFigure 2

Time from symptom onset (or test collection date if asymptomatic) to receipt of antiviral therapy (nirmatrelvir/ritonavir or intravenous remdesivir for SARS-CoV-2 and oseltamivir for influenza) for residents with SARS-CoV-2 or influenza infection (A) and separated by individual virus (B).

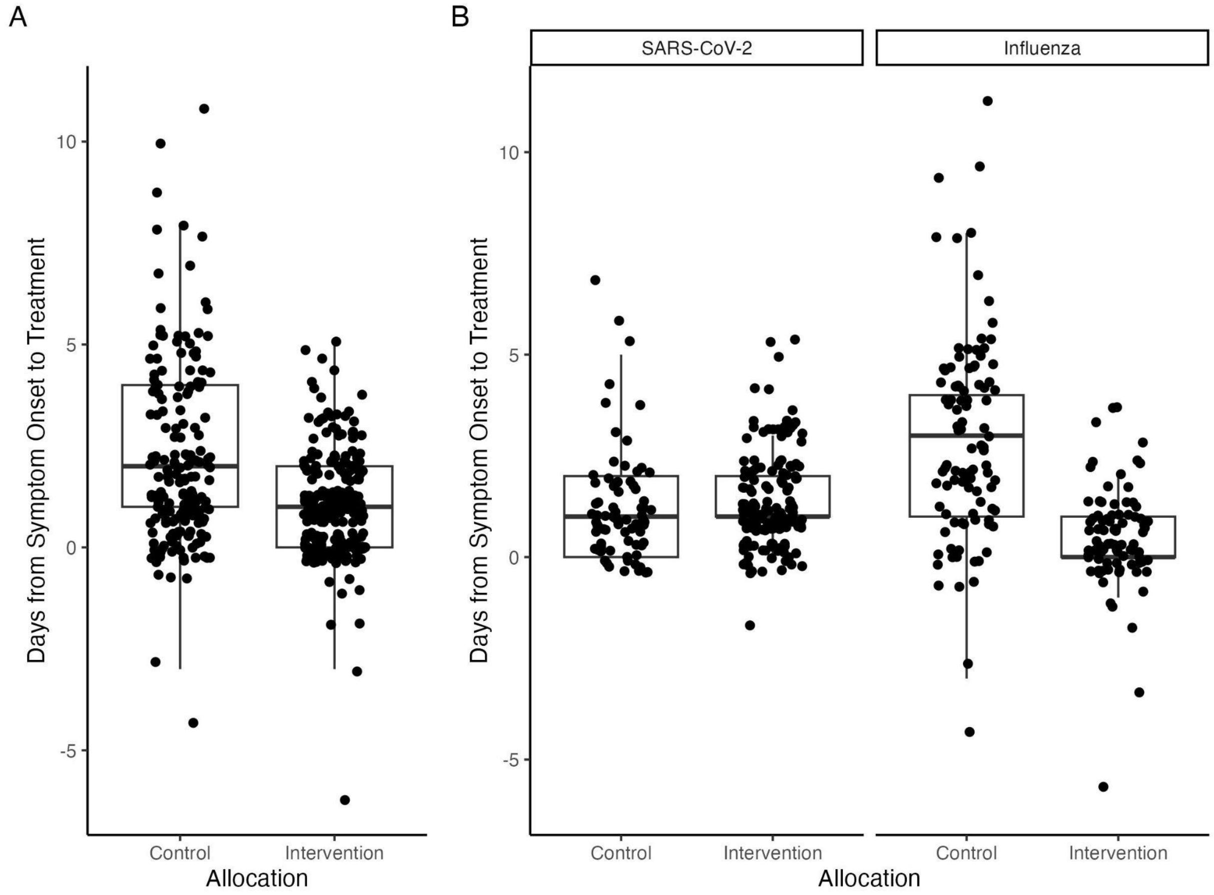

© 2026 Kandel C et al. *JAMA Internal Medicine*.
