# Supplementary material for: Respiratory Outbreak Mitigation With Point-of-Care Testing in Long-Term Care: A Randomized Clinical Trial
Source: JAMA Intern Med. 2026 Jul 6:e262644. Online ahead of print. doi: 10.1001/jamainternmed.2026.2644 (PMC13338844; doi:10.1001/jamainternmed.2026.2644)
Supplement: Supplement 4. — Data sharing statement [file jamainternmed-e262644-s004.pdf]

Data Sharing Statement

Kandel. Respiratory Outbreak Mitigation With Point-of-Care Testing in Long-Term Care. *JAMA Intern Med*. Published July 06, 2026. doi:10.1001/jamainternmed.2026.2644

Data
Additional Information: NCT06660433 Clinicaltrials.gov
https://clinicaltrials.gov/study/NCT06660433
Data available: Yes
Data types: Deidentified participant data, Data dictionary
How to access data: Deidentified participant data and the data dictionary will be available to researchers whose proposed use of the data has been approved by a qualified Research Ethics Board (REB) and by the REB of Michael Garron Hospital. Access is via christopher.kandel@tehn.ca and will be available following publication for 5 years. After approval of a proposal, data will be transferred after a signed data transfer agreement has been executed.
When available: With publication

Supporting Documents
Document types: None

Additional Information
Who can access the data: Data will be made available to researchers whose proposed use of the data has been approved.
Types of analyses: Data will be made available for any purpose.
Mechanisms of data availability: Data will be made available with investigator support, after approval of a proposal and signed data access agreement.
